# Cooperative interaction between AAG and UV-DDB in the removal of modified bases

**DOI:** 10.1093/nar/gkac1145

**Published:** 2022-12-13

**Authors:** Sunbok Jang, Namrata Kumar, Mathew A Schaich, Zhou Zhong, Barbara van Loon, Simon C Watkins, Bennett Van Houten

**Affiliations:** Department of Pharmacology and Chemical Biology, University of Pittsburgh School of Medicine, PA 15261, USA; UPMC Hillman Cancer Center, PA 15213, USA; College of Pharmacy and Graduate School of Pharmaceutical Sciences, Ewha Womans University, Seoul 03760, South Korea; UPMC Hillman Cancer Center, PA 15213, USA; Molecular Genetics and Developmental Biology Graduate Program, University of Pittsburgh School of Medicine, PA 15261, USA; Department of Pharmacology and Chemical Biology, University of Pittsburgh School of Medicine, PA 15261, USA; UPMC Hillman Cancer Center, PA 15213, USA; Department of Pharmacology and Chemical Biology, University of Pittsburgh School of Medicine, PA 15261, USA; UPMC Hillman Cancer Center, PA 15213, USA; Department of Clinical and Molecular Medicine, Faculty of Medicine and Health Sciences, Norwegian University of Science and Technology (NTNU), 7491, Trondheim, Norway; Center for Biologic Imaging, University of Pittsburgh, PA 15261, USA; Department of Pharmacology and Chemical Biology, University of Pittsburgh School of Medicine, PA 15261, USA; UPMC Hillman Cancer Center, PA 15213, USA

## Abstract

UV-DDB is a DNA damage recognition protein recently discovered to participate in the removal of 8-oxo-7,8-dihydro-2′-deoxyguanosine (8-oxoG) by stimulating multiple steps of base excision repair (BER). In this study, we examined whether UV-DDB has a wider role in BER besides oxidized bases and found it has specificity for two known DNA substrates of alkyladenine glycosylase (AAG)/N-methylpurine DNA glycosylase (MPG): 1, *N*^6^-ethenoadenine (ϵA) and hypoxanthine. Gel mobility shift assays show that UV-DDB recognizes these two lesions 4–5 times better than non-damaged DNA. Biochemical studies indicated that UV-DDB stimulated AAG activity on both substrates by 4- to 5-fold. Native gels indicated UV-DDB forms a transient complex with AAG to help facilitate release of AAG from the abasic site product. Single molecule experiments confirmed the interaction and showed that UV-DDB can act to displace AAG from abasic sites. Cells when treated with methyl methanesulfonate resulted in foci containing AAG and UV-DDB that developed over the course of several hours after treatment. While colocalization did not reach 100%, foci containing AAG and UV-DDB reached a maximum at three hours post treatment. Together these data indicate that UV-DDB plays an important role in facilitating the repair of AAG substrates.

## INTRODUCTION

Cellular DNA is prone to oxidation, deamination and alkylation from both endogenous and exogenous sources (1–3). The resulting DNA lesions are repaired through base excision repair (BER), which is initiated by one of eleven DNA damage specific mammalian glycosylases. Alkyladenine glycosylase (AAG), also known as N-methylpurine DNA glycosylase (MPG), is an interesting glycosylase that appears to recognize structurally diverse substrates. These include the alkylation products N7-methyl G and N3-methyl A, as well as 1,*N*^6^-ethenoadenine (ϵA), a product of lipid peroxidation from exposure to vinyl chloride, or chloroacetaldehyde as reviewed in (4) and finally, hypoxanthine (Hx), the deamination product of adenine.

Chronic inflammation can cause lipid peroxidation producing DNA reactive aldehydes that generate base damage including ϵA, which has been found to increase 5- to 10-fold in cancer prone human diseases such as alcoholic fatty liver, Crohn’s disease, and chronic pancreatitis (5). Since ϵA is a replication-blocking lesion, failure to remove the lesion can be cytotoxic (6,7). Hx has also been shown to increase during chronic inflammation and has been found to occur in animal tissue at a frequency of about 0.5 lesions/10^6^ deoxynucleosides but can rise ∼10-fold following a model of chronic colitis due to *Heliobacter pylori* infection in mice (8). Since Hx can pair with cytidine, it is mutagenic and has been found to cause AT to GC transition mutations in human cell lines (9).

During one branch of BER, AAG efficiently recognizes the DNA damage by flipping out the modified nucleotide into a recognition pocket. Using its N-glycosylase activity, AAG excises these damaged bases leaving a potentially cytotoxic abasic site (AP-site) (10). APE1 nicks the DNA at AP-sites leaving a 5-deoxyribose phosphate (dRP) moiety. This nick can activate PARP1, which produces poly-(ADP)-ribose chains and helps recruit the scaffold protein XRCC1, which further facilitates the recruitment of DNA polymerase β and DNA Ligase III. DNA polymerase β removes the deoxyribose moiety and fills in the nucleotide gap. Finally, a DNA ligase seals the nick and completes repair (11). Incomplete repair of alkylation damage has been shown to be toxic to cells (12–14). Unlike other glycosylases that bind more tightly to their abasic site product, AAG would appear to have equal to or lower affinity for abasic sites than either ϵA or Hx moieties (15,16). Our previous work using biochemical, single molecule and cellular studies have demonstrated a direct role of UV-DDB (UV-damaged DNA-binding protein ) in processing 8-oxoG lesions stimulating OGG1, MUTYH and APE1 activities (17,18). Recently, Thoma has shown that UV-DDB has the ability to bind to abasic sites in reconstituted nucleosomes and change their register as much as 3 bp, thus making the lesion more accessible to repair (19).

UV-DDB is a heterodimeric protein consisting of DDB1 (127 kDa) and DDB2 (48 kDa). UV-DDB is part of a larger complex containing cullin-4A/4B and RBX1 that possess E3 ligase activity. UV-DDB ubiquitinates histones to destabilize the nucleosome, thereby allowing downstream repair proteins to access the lesion (20,21). These results suggest that UV-DDB may play a damage sensor role during BER by interacting with specific types of base damage contained in nucleosomes and stimulating the activity of damage specific glycosylases. We were therefore interested in determining whether other glycosylases, such as AAG might be stimulated by UV-DDB.

In this study, we found that UV-DDB recognizes alkylated bases efficiently and binds specifically to DNA substrates containing ϵA and hypoxanthine Hx moieties. UV-DDB was also found to stimulate AAG activity on both these DNA substrates by ∼4- to 5-fold. Single molecule and native gel studies revealed that UV-DDB can form complexes with AAG on DNA and facilitate its dissociation. Finally, we have found that treatment of U2OS cells with methyl methanesulfonate (MMS) produced discrete foci accumulating both DDB2 and AAG, some of which co-localize. This study thus helps expand the role of UV-DDB as a universal damage sensor in initial damage recognition of BER pathway.

## MATERIALS AND METHODS

### Expression and purification of recombinant UV-DDB, AAG WT and AAG D80 EQ

Recombinant full-length UV-DDB (DDB1-DDB2 heterodimer) was expressed in Sf9 cells coinfected with recombinant baculovirus of DDB1-His6 and DDB2-Flag, as performed previously (17). Briefly, DDB1-His6 and DDB2-Flag were purified using a 5 ml His-Trap HP column pre-charged with Ni2+ (GE Healthcare) and anti-FLAG M2 affinity gel (Sigma). The pooled anti-FLAG eluates were size fractionated on a HiLoad 16/60 Superdex 200 column (Amersham Pharmacia) in UV-DDB storage buffer (50 mM HEPES (4-(2-hydroxyethyl)-1-piperazineethanesulfonic acid), pH 7.5, 200 mM KCl, 1 mM EDTA, 0.5 mM PMSF (phenylmethylsulfonyl fluoride), 2 mM DTT (dithiothreitol), 10% glycerol,and 0.02% sodium azide). Purified fractions of DDB1-DDB2 complex from the Superdex200 were aliquoted and flash frozen with liquid nitrogen and stored at −80°C. AAG WT was purchased from NOVUS (Saint Charles, MO) and AAG Δ80 p.E125Q (EQ) was purified as previously described (22).

### DNA substrate preparation

#### 37 bp DNA duplexes for excision assay and native gel experiment

The following oligonucleotides sequences (X = ϵA, Y = Hx and Z = Tetrahydrofuran) were used:

ϵdA37-top: 5′-CCG AGT CAT TCC TGC AGC G**X**G TCC ATG GGA GTC AAA T-6FAM-3′

ϵdA37-bottom: 5′-A TTT GAC TCC CAT GGA CTC GCT GCA GGA ATG ACT CGG-3′

Hx37-top: 5′-CCG AGT CAT TCC TGC AGC G**Y**G TCC ATG GGA GTC AAA T-6FAM-3′

Hx37-bottom: 5′-A TTT GAC TCC CAT GGA CTC GCT GCA GGA ATG ACT CGG-3′

THF37-top: 5′-CCG AGT CAT TCC TGC AGC G**Z**G TCC ATG GGA GTC AAA T-6FAM-3′

THF37-bottom: 5′-A TTT GAC TCC CAT GGA CTC GCT GCA GGA ATG ACT CGG-3′

The 37 bp duplex containing ϵA was prepared by annealing ϵdA37-top (purchased from Midland, USA) and ϵdA37-bottom (purchased from IDT, USA). Hx37 was prepared by annealing Hx37-top (purchased from Midland, USA) and Hx37-bottom (purchased from IDT, USA). THF37 was prepared by annealing THF37-top and THF37-bottom (all purchased from IDT, USA). Annealing reactions were done at 95°C for 5 minutes in 10 mM Tris-HCl, pH 8.0 and 100 mM KCl and then cooled to room temperature slowly for 4 h by turning off the heating device.

#### Defined lesion plasmids

Plasmids containing single site-specific tetrahydrofuran (THF) adducts were prepared as described previously (23). Briefly, purified pSCW01 plasmids were nicked by *Nt.BstNBI* to create a 37-base gap. A 37mer containing a single abasic site (THF37-top, above) was annealed into this gap and the backbone was sealed with T4 DNA ligase. The THF arrays were prepared using the defined lesion plasmid described above. Lesion-containing pSCW01 was linearized via restriction digest by *XhoI* (NEB) then incubated with T4 DNA ligase (NEB) to achieve long (>40 kbp) tandemly ligated products with one THF site every 2 kb.

### AAG excision assay

To measure the removal of base lesion (ϵdA or Hx) from duplex DNA, we used an excision assay. Reactions were carried out in a volume of 10 μl containing AAG excision buffer (20 mM HEPES, pH 7.9, 50 mM NaCl, 1 mM MgCl_2_, 1 mM DTT, 0.1 mg/ml of BSA), 50 nM of fluorescein-labeled ϵA or Hx containing duplex DNA and the indicated amount of AAG WT and UV-DDB. The concentrations chosen produced around 20% excision in the absence of UV-DDB, allowing the remaining 80% to be excised upon UV-DDB stimulation. Reactions were incubated at 37°C for each time point (up to 3 h) and rapidly quenched by adding an equal volume of gel loading buffer (2× formamide dye solution), heated at 95°C for 5 min, then cooled on ice for 5 min. About 0.1 M NaOH was included in the quenching step to induce DNA nicking at the abasic site. The reaction product was separated by electrophoresis on 10% denaturing polyacrylamide gel and visualized using a laser scanner for fluorescence (Typhoon, Amersham). The substrate and product bands were quantified using ImageJ.

### Equilibrium dissociation constant determination from electrophoretic mobility shift assay (EMSA)

To determine the equilibrium dissociation constants of UV-DDB binding to various DNA lesions we conducted electrophoretic mobility shift assays with fluorescein-modified DNA substrates. DNA substrates, held constant at 8 nM, were mixed with increasing amounts of UV-DDB and incubated for 20 min at room temperature in reaction buffer (20 mM HEPES, pH 7.5, 150 mM NaCl, 2 mM DTT, 5% glycerol, 0.5 mg/ml BSA). A 5 μl aliquot of each reaction was loaded on a 5% polyacrylamide (37.5:1, acrylamide: bis) native gel, in duplicate, and run at 100 V for 50 min at 4°C in 1/2X TBE (Tris Borate EDTA). Gels were imaged using a laser scanner for fluorescence (Typhoon, Amersham).

Gel images were quantified by measuring signal intensity of each band (ImageJ, NIH). The percentage of DNA bound was determined by dividing the intensity of the shifted (‘bound’) DNA by the sum of all bands in a lane. Background signals from blank regions of the gel were subtracted from the signal intensities obtained from bands. These values were plotted against UV-DDB concentration and the data were fit to the following equation via nonlinear regression in GraphPad Prism:

$$\begin{eqnarray*} && \% {\rm DNA}\ {\rm Bound }= {\rm{ }}100{\rm{ }} \nonumber \\ && \quad \times {\rm{ }}\frac{{\left( {D + P + {K}_{\rm d}} \right) - \sqrt {{{\left( {D + P + {K}_{\rm d}} \right)}}^2 - 4DP} }}{{2D}} \end{eqnarray*}$$

where *K*_d_ is the equilibrium dissociation constant, *P* is the total protein concentration, and *D* is the total DNA concentration, all in units M. This model was chosen because our experimental conditions required that the DNA concentration be in the same molar range as the *K*_d_ (24).

### Native PAGE

AAG-DNA reaction was prepared by combining 8 nM of 37 bp THF DNA with 60 nM of AAG in reaction buffer (20 mM HEPES, pH 7.5, 150 mM NaCl, 5 mM DTT, 0.5 mg/ml BSA and 5% glycerol) and incubated for 10 min at RT, then were mixed with increasing amounts of UV-DDB (0–64 nM) in a final reaction volume of 10 μl. Each reaction was incubated for 30 min at RT then immediately loaded on two pre-run 5% polyacrylamide (37.5:1, acrylamide: bis) native gels and run at 100 V for 50 min at 4°C in 0.5X TBE (4.5 mM Tris, 44.5 mM boric acid, 1 mM EDTA, pH 8.4). DNA bands were visualized using a laser scanner for fluorescence (Typhoon, Amersham). The percentage of total DNA bound by each protein was determined by measuring the band intensity present in the bound states and dividing by the total band intensity in the lane. Background signals from blank regions of the gel were subtracted from the signal intensities obtained from bands. The percentage of DNA bound in each reaction was plotted against the concentration of UV-DDB.

### DNA tightrope assay

Single-molecule DNA tightrope assay was performed as described previously (23,25,26). Briefly, poly-l-lysine (Wako Pure Chemicals) coated silica beads (5 μm, Polysciences Inc.) were deposited onto a PEG-treated coverslip (24 × 40 mm; Corning) in a custom flow cell. Defined lesion (abasic site) substrates were strung up across the beads via hydrodynamic flow. DNA substrates were made by tandem ligation of pSCW01 plasmid (∼2 kb) with a single, site specific THF modification and proximal biotinylated thymine.

#### Protein labeling

Prior to imaging, purified His-tagged AAG was labeled with secondary antibody-coated 605 nm quantum dots (Qdots; Invitrogen) through α-His primary antibody (Qiagen). For UV-DDB-facilitated dissociation experiments, quantum dot labeled AAG was injected into the flow cell at final concentrations of 2.6 nM with 1 × UV-DDB conjugated to goat α-Flag primary antibody. For colocalization experiments, purified UV-DDB was conjugated to streptavidin coated 705 nm Qdots through biotinylated goat α-Flag primary antibody (Bethyl), and quantum dot labeled AAG or UV-DDB were injected into the flow cell at final concentrations of 2.6 or 3.1 nM, respectively. We conjugated each protein in separate reaction tubes and injected into the flow cell separately for dual-color experiments; flow was stopped during the observation period. Furthermore, we performed a control experiment to check whether there is any unwanted interaction between 705Qdot-labeled UV-DDB (DDB1 is His-tagged) and α-His primary antibody conjugated to 605Qdots. We injected 705Qdot conjugated UV-DDB into the flow cell and then injected 605Qdot conjugated with α-His primary antibody and observed for 4 h. During this time, 20 particles were recorded, but none were co-localized. All binding experiments were carried out in tightrope buffer (25 mM HEPES, pH 7.5, 150 mM NaCl, 0.1 mg/ml BSA (Roche), 50 nM biotin, and 1 mM DTT).

#### Data collection

Labeled proteins were visualized using oblique angle fluorescence (Nikon Eclipse Ti inverted microscope with Nikon 100X TIRF objective and 1.45 numerical aperture) with a 488 nm laser (power 1–2 mW) at the back focal plane to excite Qdots and the appropriate emission filter (Chroma) applied at RT. Movies were taken for 5 min with frame rates between ∼11 and 12.5fps.

#### Data analysis

Images were acquired using Nikon Elements (4.2) and exported as TIFF stacks for kymograph processing and analysis in ImageJ (NIH). Differences between dissociation rates and motility fractions were assessed by one-way ANOVA. The mean squared displacement (MSD) was calculated for all motile phases using custom scripts in MATLAB:

$$\begin{equation*}MSD \left( {n\Delta t} \right) = \frac{1}{{N - n}} \mathop \sum \limits_{i = 1}^{N - n} {\left( {{x}_{i + n} - {x}_i} \right)}^2\end{equation*}$$

where *N* is total number of frames in the phase, *n* is the number of frames at a given time step, *Δt* is the time increment of one frame, and *x_i_* is the particle position in the *i*th frame (27). The diffusion coefficient (*D*) was determined by fitting a linear model of one-dimensional diffusion to the MSD plots:

$$\begin{equation*}MSD \left( {n\Delta t} \right) = 2D{\left( {n\Delta t} \right)}^\alpha + y\end{equation*}$$

where *y* is a constant (*y*-intercept). Fittings resulting in *R^2^* < 0.8 or using <10% of the MSD plot were not considered.

### Single-molecule analysis of DNA-binding proteins from nuclear extracts (SMADNE)

#### Nuclear extract generation and characterization

Nuclear extract protocols used for SMADNE as in (28) and as described below. Dulbecco’s Modified Eagle Medium (DMEM) supplemented with 4. 5g/l glucose, 10% fetal bovine serum (Gibco), 5% penicillin/streptavidin (Life Technologies) was used to culture U2OS cells for making nuclear extracts at 5% oxygen. About 4 μg of GFP-AAG plasmid were added to 4 million cells to transfect using the lipofectamine 3000 reagent and protocol for 24 h as per the manufacturer’s instruction (Thermo Fisher). Nuclear extraction was performed the day after transient transfection with a commercially available nuclear extract kit (Abcam). Extracts were then aliquoted into single-use aliquots and flash-frozen in liquid nitrogen prior to storage at −80°C. Upon use for single-molecule experiments, nuclear extracts were immediately diluted after thawing, at a ratio of 3:10, into tightrope buffer with biotin excluded, but 1 mM Trolox included to increase the photostability of GFP. Concentrations of GFP-AAG were determined by fitting the background pixel intensities to a standard curve previously generated with purified GFP.

#### DNA substrate generation

Lambda DNA for nuclear extract experiments on the LUMICKS C-trap was purchased from New England Biolabs. Biotin tags was added to the DNA by adding a mix of 6 μg lambda DNA, 50 μM nucleotide mix (with dATP, dGTP, dTTP and biotinylated dCTP), 15 units of Klenow fragment polymerase (NEB) and 1x concentration of NEB Buffer 2. The reaction was incubated for 30 min at 37°C and then the free nucleotides were removed from solution via ethanol precipitation, with 1 μg/μl glycogen used as a co-precipitant to increase the yield. DNA containing Hx and Cy3 fiducial markers of the damage positions was generated by first treating 1 μg of DNA with the nickase Nt.BspQI (NEB) to generate 10 nicks in lambda DNA at specific sites. Two of the positions are close together and not resolved in our assay and another is too close to the bead to be observed so only 8 sites are observed. After nicking the DNA, fluorescent nucleotides were incorporated using nick translation for identification of nick sites, using a 40 μM mix of dGTP, dCTP, dITP (deoxyinosine triphosphate, the nucleotide form of hypoxanthine) and Cy3-labeled dUTP, in the presence of 10 units of pol I and 800 ng nicked lambda DNA.

#### Data collection and analysis

Nuclear extract data was collected on a LUMICKS C-trap. After passivating the flow cell 4.4 um polystyrene beads coated in streptavidin were trapped in channel 1 with two optical traps and a single DNA was captured between streptavidin coated beads in channel 2. When a DNA strand was caught, its force-distance curve was compared to a model using the extensible worm-like chain model to ensure that only one piece of DNA was tethered to the beads. The DNA was briefly washed in degassed tightrope buffer without biotin but with 1 mM Trolox included while opening the valve to flow channel 4, containing the nuclear extracts with overexpressed GFP-AAG. The flow was then stopped, and the tethered DNA moved to the extracts and pulled to a tension of 10 pN—enough tension to accurately position the DNA without overstretching it. The positions of the DNA, as determined by the fiducial Cy3 labels, were scanned in kymograph mode at 10–30 frames per second. Laser powers used were 2 μW for the 488 nm laser and 2.5 μW for the 562 nm laser. All data was collected with a 1.2 NA 60× water emersion objective and emitted photons measured with single-photon avalanche photodiode detectors with specific band pass filters for 500–550 nm and 575–625 nm band pass filter. After a brief (<5 s) exposure to the 562 nm laser to determine the positions of the fiducial markers, the laser was toggled off to collect with only the 488 nm laser (thus minimizing the excitation of the Cy3 dye while exciting the GFP-AAG).

Measurements of GFP photostability were taken by applying continuous exposure to proteins fixed to the bottom of the flow cell. Photon counts were binned by each second of exposure and fit to a single-exponential decay function. GFP lifetime was 40.4 ± 6.3 s at 10 frames per second, so that rate was used to correct binding lifetimes for photobleaching. The correction for photobleaching increased the AAG lifetime by 0.2 s. Kymographs were analyzed with custom software from LUMICKS (Pylake). For visualization of the kymographs and 2D scans after exporting, the utility C-Trap .h5 Visualization GUI (2020) by John Watters was used as downloaded from harbor.lumicks.com. Line tracking was performed using a custom script from LUMICKS based on a Gaussian fit over the line to determine its positions (29). To correct for GFP blinking, any two lines that appeared at the same position with <2 s of gap time between them were manually connected. MSD analysis was performed also using Pylake and fit to the equation and parameters described above with the tightrope data.

### Cell treatment and imaging

For cell survival experiments, siRNA to either AAG or DDB2 was transfected into U2OS cells (300 000 cells/ well of a 6-well mm dish) and after 48 h were treated with treated with methyl methanesulfonate (MMS) (0–0.5 mM) for 1 h. The cells were replated at 100 cells in each well of a 6-well plate in triplicate. Eight days later, the cells were stained, and the colonies were counted. Western analysis revealed robust knockdown (KD) continuing for the length of the experiment (see Figure 7A). siRNAs used: siControl: 5′AATTCTCCGAACGTGTCACGT-3′; siDDB2: 5′-CAA CUA GGC UGC AAG ACU U-3′; siAAG: 5′-CAACCGAGGCATGTTCATGAA-3′.

Initial experiments of DDB2 imaging were done with transient transfection of U2OS cells with DDB2-mCherry. Forty-eight hours post transfection, cells were treated with MMS (0.5 or 1 mM) MMS for 30 min and fixed for immunofluorescence. Cells were stained for mCherry (1:250; Abcam #ab167453) and y-H2AX (1:100, Santacruz #sc-517348). Secondary antibodies used: Donkey anti-mouse Alexa488 (1:1000; Thermo Fisher Scientific #A21202), Goat anti-Rabbit Alexa-594 (1:1000; Thermo Fisher Scientific #A11012).

U2OS cells stably expressing mNeonGreen-DDB2 (∼3× over endogenous levels) were plated in a MatTek dish overnight. The next day cells were incubated with DMEM medium containing 2 mM MMS for 60 min 37°C 5% CO_2_ incubator. The media was aspirated and replaced with fresh medium three times and the cells were allowed to recover for 0, 1, 3, 12 and 24 h. The cells were fixed and stained with mouse primary anti-AAG (1:100; Novus Biologicals #H00004350-M04) for 1 h at room temperature and goat anti-mouse Alexa 594 (1:1000; Thermo Fisher Scientific #A-11005) for 45 min at room temperature. Cells were washed with 1X PBS twice before mounting. Cells were pre-extracted using CSK buffer by incubating on ice for 2 min before fixation.

Imaging of slides was performed either the Nikon Ti inverted microscope with a 63× objective (1.4 NA) using a z stack of 0.2 μm or at the Center for Biologic Imaging (Simon Watkins) with A1 confocal microscope with 3D stacks at 0.2 μm Z step for a total of 5 μm. The exposure time of each channel was kept consistent throughout samples. Images were analyzed using the NIS Elements advance research software (5.2 or GA3).

## RESULTS

**Figure 1.**
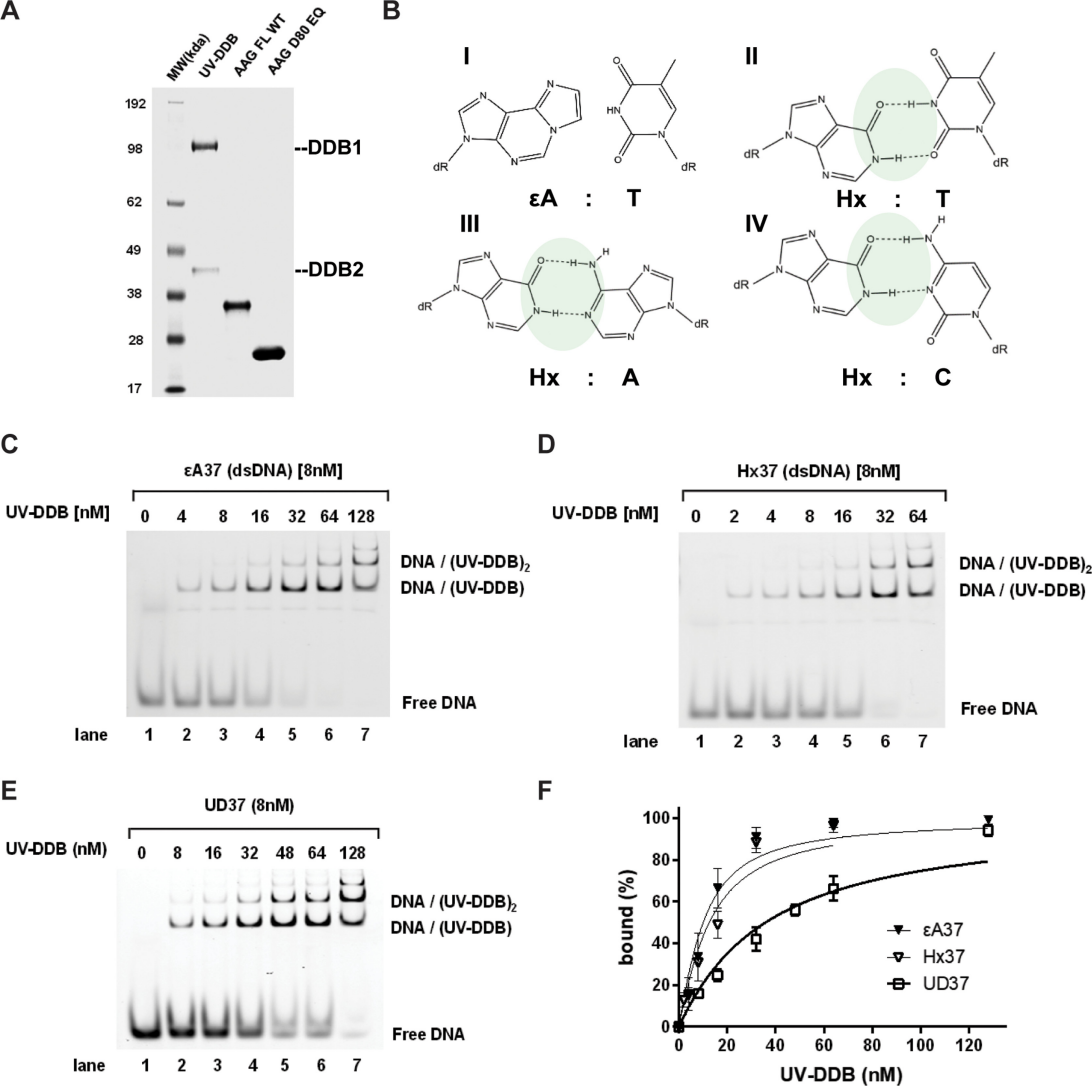
Purified proteins, substrate chemical structure, and UV-DDB EMSA experiments. (**A**) Coomassie stain of SDS-PAGE showing purified proteins used in this study. (**B**) Chemical structure of ϵA paired with thymine and Hx paired with paired with thymine, adenosine, and cytosine. (**C–E**) Representative native gels for EMSA experiments, quantification and binding isotherm fitting shown in F. UV-DDB was mixed with different 37bp fluorescein-labeled dsDNA substrates with different lesions/modifications, as indicated. (**F**) Binding isotherms for ϵA, Hx and undamaged (UD) DNA. Data is plotted as mean ± SEM of three independent experiments.

Previous studies have suggested that UV-DDB in addition to binding tightly to abasic sites is capable of recognizing 8-oxoG:C and 8-oxoG:A pairs in DNA duplexes (17). These data suggest that UV-DDB has a broader substrate recognition repertoire than only UV-induced photoproducts. We therefore tested as to whether UV-DDB recognizes ϵA and Hx damage. Using gel mobility shift assays, with increasing amounts of UV-DDB and fixed amounts of a 37 bp duplex containing either ϵA:T or Hx:T pairs, we found that UV-DDB bound to these substrates with equilibrium dissociation constants, *K*_d_, of 6.0 ± 0.7 nM or 8.6 ± 0.7, respectively, which is considerably lower affinity as compared to AAG (30) (Figure 1 and Table 1). These values are close to UV-DDB’s affinity for a 37 bp fragment containing a TT cyclobutane pyrimidine dimer (*K*_d_ = 4.5 nM) (31). In these experiments UV-DDB was found to bind to undamaged DNA about 5- to 6-fold less efficiently, with a *K*_d_ of 31.8 ± 2.0 nM.

**Table 1.** Apparent equilibrium dissociation constant (*K*_d_) of UV-DDB to different kinds of DNA lesions, related to Figure 1

Substrate	*K*_d_ UV-DDB [nM]	*K*_d_ AAG [nM]
THF	1.8 ± 0.4^a^	
CPD	4.5 ± 0.3^b^	
ϵA	6.0 ± 0.7	0.029 ± 0.003^c^
Hx	8.6 ± 0.7	0.390 ± 0.061^c^
undamaged	31.8 ± 2.0^a^	

Apparent equilibrium dissociation constants (*K*_d_) were determined from data presented in Figure 1F. These data are based on three independent experiments were performed, respectively. The *K*_d_ value is the best-fit value to the three points ± the S.E. of the fit to the observed data.

^a^: Binding affinity to TFH containing substrate varies with UV-DDB preparation. We routinely see binding affinities between 1–3 nM for THF and 32–42 nM for undamaged DNA in the absence of Mg2+

^b^: from Beecher *et al.* DNA Repair, 2020 (31).

^c^: from Lee *et al.* Biochemistry, 2009 (30).

### UV-DDB stimulates AAG excision activity

**Figure 2.**
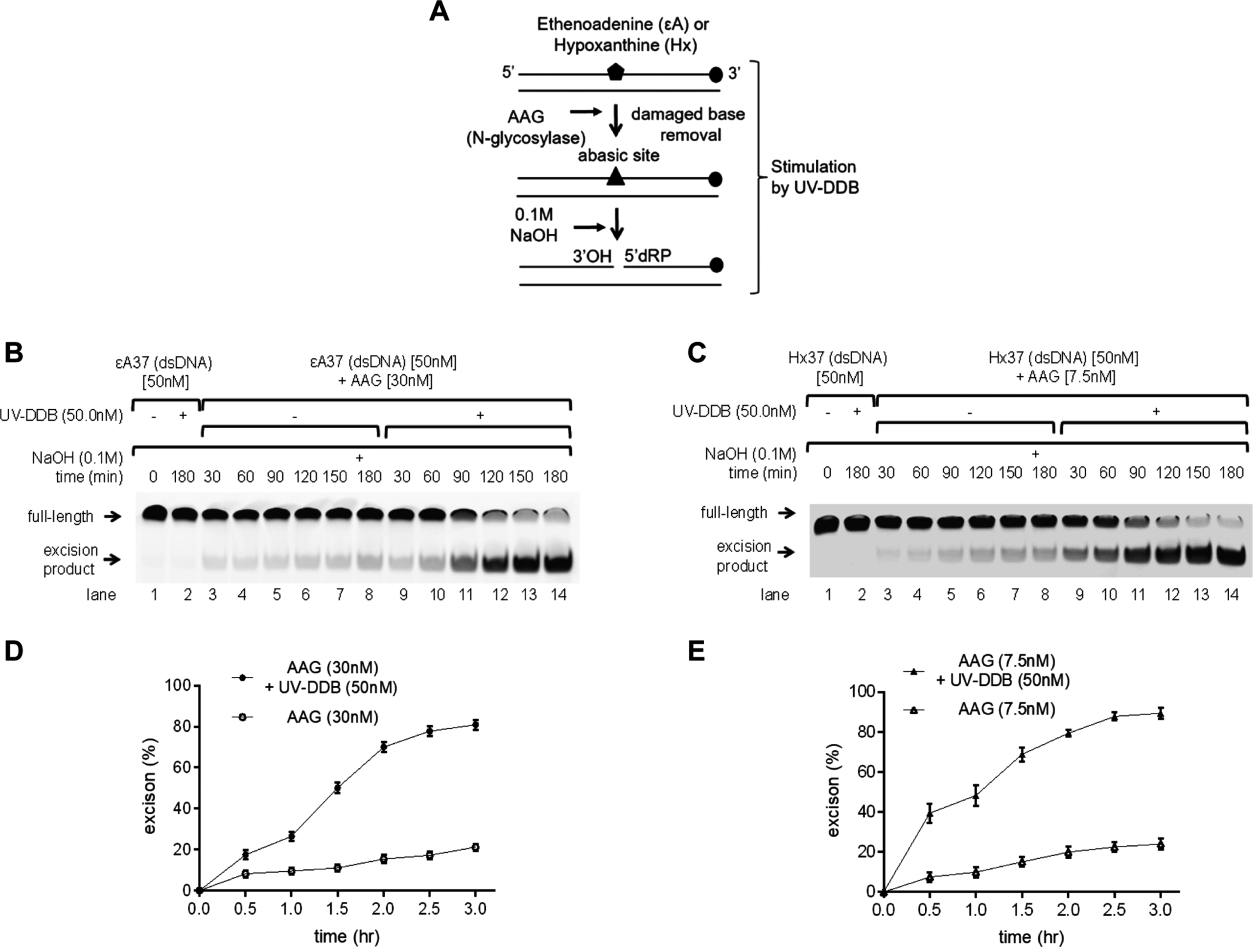
UV-DDB stimulates AAG activity. (**A**) Schematic representation of the DNA substrate containing ϵA or Hx and the proposed reaction scheme. (**B**) Stimulation of AAG excision kinetics by UV-DDB. AAG (30 nM) was incubated with dsDNA (50 nM) containing ϵdA in the absence (−) or presence (+) of UV-DDB (50 nM) at 37°C. Aliquots were withdrawn at each time point and analyzed on a 10% denaturing polyacrylamide gel. Positions of the non-cleaved full-length substrate and excised product are indicated by arrows. (**C**) Stimulation of AAG excision kinetics by UV-DDB. AAG (7.5 nM) was incubated with the hypoxanthine (Hx) dsDNA (50 nM) containing Hx in the absence (−) or presence (+) of UV-DDB (50 nM) at 37°C. Aliquots were withdrawn at each time point and analyzed on a 10% denaturing polyacrylamide gel. Positions of the non-cleaved full-length substrate and excised product are indicated by arrows. (**D**) Quantification of the stimulation of AAG excision kinetics by UV-DDB in (B). Excision product formation was quantified using ImageJ software. The excision percentage was plotted as mean ± SD. from three independent experiments, each run on duplicate gels. (**E**) Quantification of the stimulation of AAG excision kinetics by UV-DDB in (C). Quantification of the excision product formation was performed using ImageJ software. The excision percentage was plotted as mean ± SD from three independent experiments, each analyzed on duplicate gels.

Since UV-DDB shows affinity for AAG’s substrates, we asked whether UV-DDB can stimulate the enzymatic activity of AAG. To determine the effect of UV-DDB on AAG-catalyzed ϵA or Hx paired with T, a limiting amount of 30 or 7.5 nM AAG was incubated with ϵA37 or Hx37 duplex oligonucleotide (50 nM), respectively, in the absence or the presence of UV-DDB (50 nM) up to 4 h. In this assay, the AAG-processed DNA was treated with 0.1 M NaOH to convert AP sites to nicks before denaturing PAGE. A 4- to 5-fold stimulation of AAG activity was observed in the presence of UV-DDB (50 nM) (Figure 2). We also discovered that stimulation of AAG excision activity by UV-DDB was concentration dependent, reaching a maximum at 50 nM, then achieving a plateau in the cleavage reaction at higher concentration (Supplementary Figure S1). Purified UV-DDB by itself showed no detectable DNA glycosylase activity on ϵA 37 or Hx 37 (lane 2, Figure 2B–E). Finally, while APE1 (100 nM) is capable of helping turnover and stimulate AAG, UV-DDB (50 nM) increased the activity and turnover of AAG even in the presence of APE1 (Supplementary Figure S2). These findings clearly indicate that UV-DDB may promote the turnover of AAG activity or facilitate product release by working with APE1 stimulating AAG catalytic cycle during BER. The involvement of UV-DDB in AAG stimulation was further demonstrated by native gel and single molecule tightrope assay, described below.

### UV-DDB forms a complex with AAG and facilitates dissociation of AAG from abasic DNA

**Figure 3.**
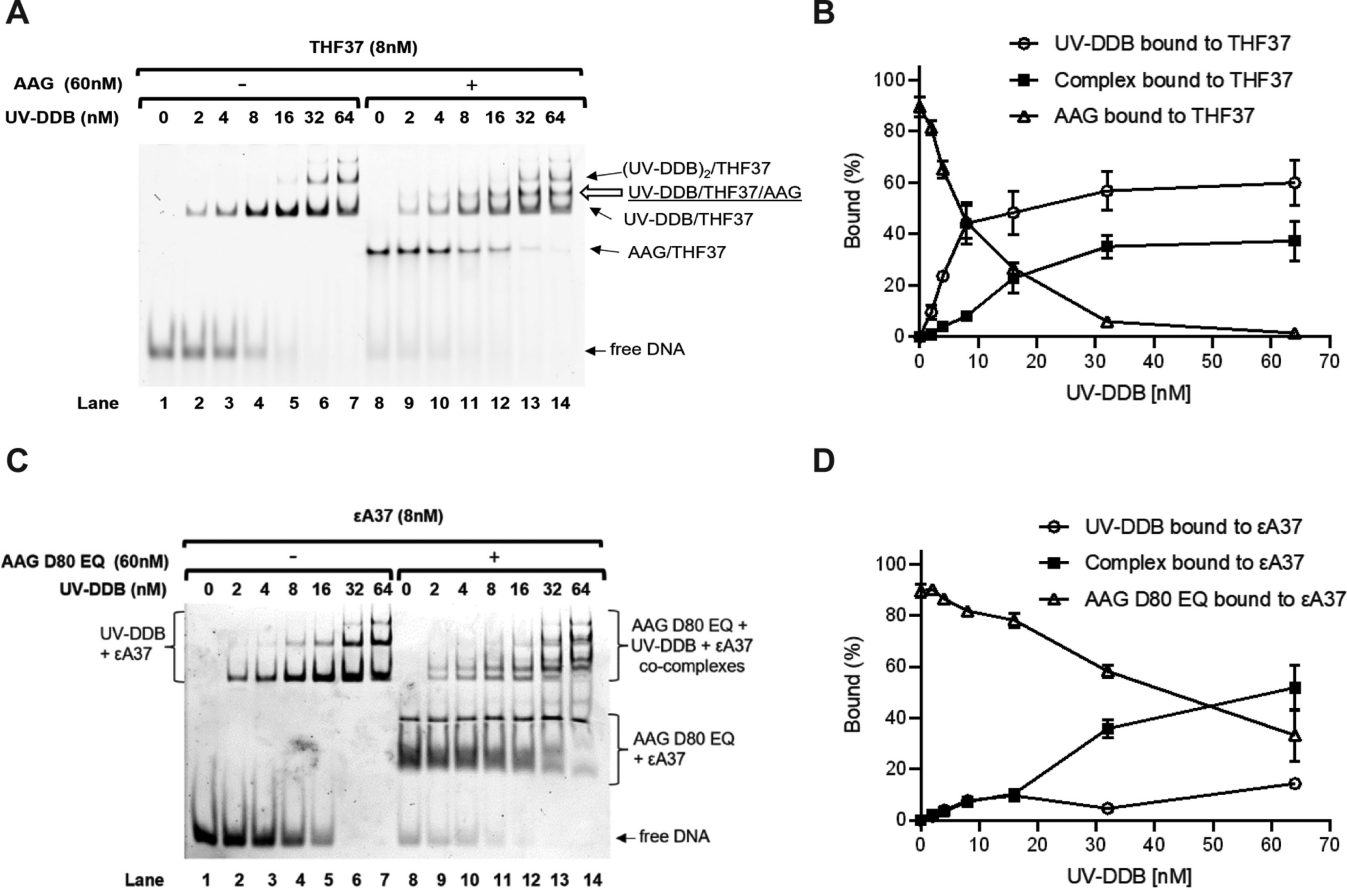
UV-DDB forms a complex with and helps turnover AAG from abasic or ϵA DNA. (**A**) Displacement and complex formation of AAG on abasic sites by UV-DDB, shown by EMSA. Binding reactions of THF37 and increasing amounts of UV-DDB with or without AAG were separated by native PAGE. Protein–DNA complexes were identified based on band migration and labeled accordingly. Representative gel shown, *N* = 3. (**B**) Quantification of (A) from lanes 8 to 14. Bound percent of total DNA by UV-DDB and/or AAG are plotted as a function of UV-DDB concentration. Data shown as the mean of three experiments ± SD. (**C**) Displacement and complex formation of AAG D80 EQ on ϵA sites by UV-DDB, shown by EMSA. Binding reactions of ϵdA37 and increasing amounts of UV-DDB with or without AAG D80 EQ were separated by native PAGE. Protein–DNA complexes were identified based on band migration and labeled accordingly. Representative gel shown, *N* = 4. (**D**) Quantification of (C) from lanes 8 to 14. Bound percent of total DNA by UV-DDB and/or AAG D80 EQ are plotted as a function of UV-DDB concentration. Data shown as the mean of four experiments ± SD.

It is well known that AAG remains sequestered by the resulting AP site following excision of ϵA or Hx bases (15). Our previous study had suggested that UV-DDB facilitates the dissociation of OGG1, MUTYH or APE1 molecules on tetrahydrofuran (THF)-containing DNA thus stimulating product release rates, which may contribute to increasing their rates of turnover (17,18). We sought to investigate this hypothesis using native PAGE. AAG WT or D80 EQ (a catalytically dead mutant) (60 nM) -THF37 duplex DNA (8 nM) complex was pre-formed by incubating these two components for 10 min at room temperature (RT). This AP site analogue (THF) was chosen because it forms stable complex with AAG WT or catalytically inactive AAG D80 EQ. THF contains a closed sugar ring and is resistant to spontaneous cleavage. Upon incubation of AAG with a THF containing 37 bp duplex, >85% of the DNA was in the form of a complex with AAG (Figure 3A and C, lane 8). Subsequently, increasing amounts of UV-DDB (0–64 nM) was added in the absence of AAG (lanes 1–7) or presence of AAG (lanes 8–14). After a 30-min incubation at RT, UV-DDB can occupy the abasic (THF) site liberated after AAG spontaneously dissociates from these sites (Figure 3A and B) or liberated AAG from complexes with UV-DDB, empty arrow in Figure 3A. These findings suggest that UV-DDB stimulates AAG activity by preventing AAG’s reassociation to its product which can allow the recycling of the AAG and therefore inducing stimulation of AAG to react with other substrates.

**Figure 4.**
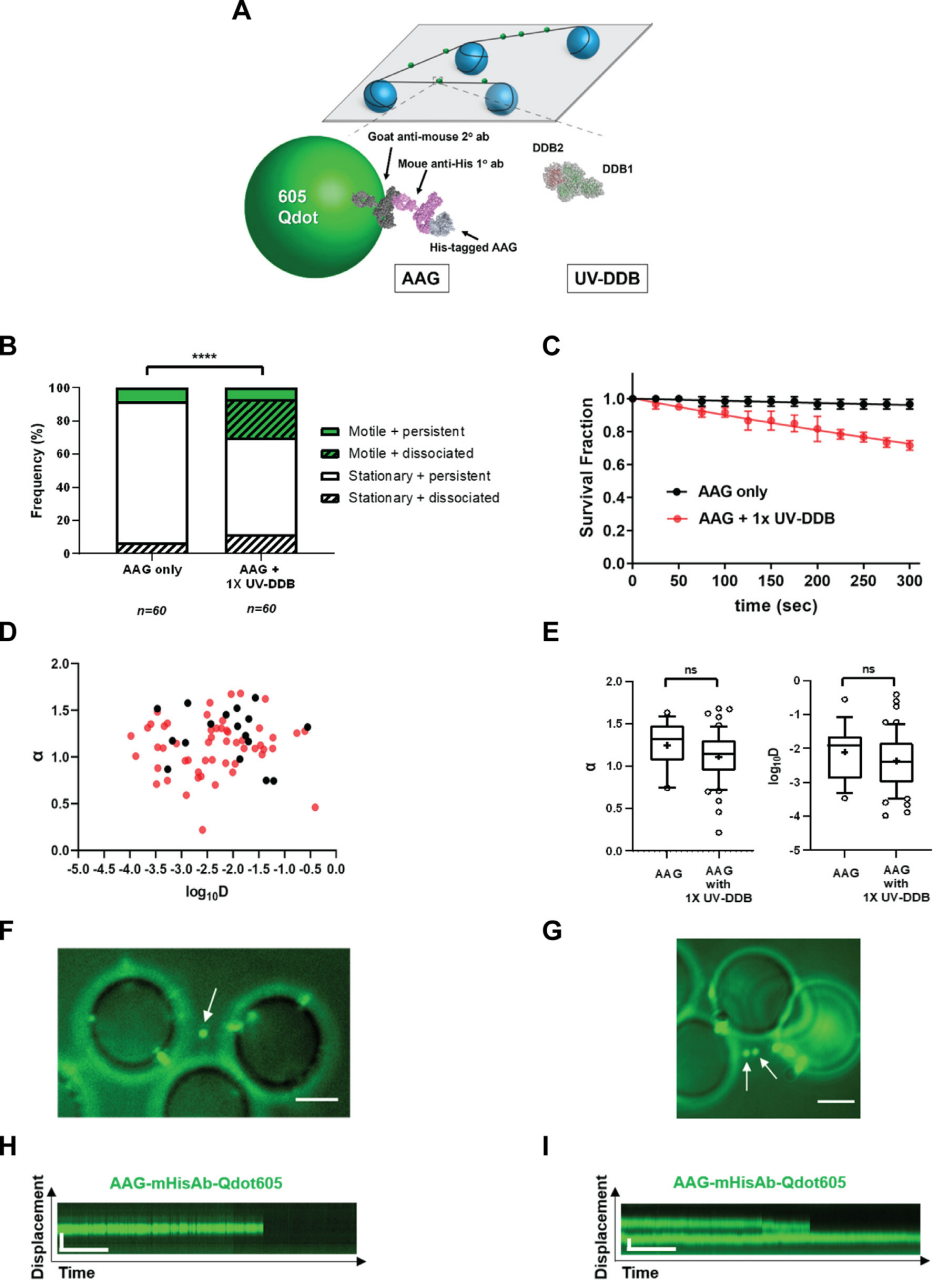
Single molecule analysis reveals that UV-DDB stimulates AAG by facilitated mobility or dissociation. (**A**) Experimental design of DNA tightrope assay to study UV-DDB induced mobility and dissociation of AAG. (top) Long DNA substrates with defined abasic sites (THF) every 2 kb are suspended between silica beads (bottom, left) His-tagged AAG is labeled with primary mouse-anti-His antibody and secondary goat-anti-mouse antibody conjugated to a 605 nm Qdot. (bottom right) unlabeled UV-DDB. Qdots are shown as a model and are not to scale. (**B**) Stack bar graph showing the fraction of motile (green) versus stationary (white) and persistent (solid) versus dissociating (diagonal lines) particles of 605Qdot labeled AAG in the absence (-) or presence (+) of un-labeled 1x UV-DDB on abasic (THF) DNA during 300s observation. (****, *P* < 0.0001 by χ2 test). (**C**) Effects of UV-DDB on the lifetimes of AAG-DNA complexes. Data plotted as the mean ± SEM from three independent experiments. For each condition, survival fraction decay is fit to a single exponential decay function to obtain the half-life. (**D**) Anomalous diffusion exponent (α) versus diffusion coefficient (log10D) plotted for AAG (black filled circles) and AAG with 1x UV-DDB (red filled circles). (**E**) Box and whisker plot (10–90 percentile) of left, the anomalous diffusion exponent (α) and right, the diffusion coefficient (log10D) calculated for AAG only (*n* = 17 phases) and AAG with 1X UV-DDB (*n* = 53 phases) phases on long DNA substrates with defined abasic sites (THF) every 2 kb. +, sample mean, *P < 0.1, ns, not statistically signficant by two-tailed Student's *t* test. (**F**) Image of 605Qdot-labled AAG (green) on abasic (THF) tightrope suspended between beads in the 1x presence of un-labeled UV-DDB. Scale bar represents 2.5 μm. Arrow points to non-motile dissociated AAG particle. (**G**) Kymograph of 605Qdot-labeled AAG (green) with non-motile and dissociated particle (**F**). Horizontal and vertical scale bars represent 50 s and 2 kb, respectively. (**H**) Image of 605Qdot-labled AAG (green) on abasic (THF) tightrope suspended between beads in the 1x presence of un-labeled UV-DDB. Scale bar represents 2.5 μm. Arrow points to motile or non-motile dissociated AAG particles. (**I**) Kymograph of 605Qdot-labeled AAG (green) with motile or non-motile dissociated particle (**H**). Horizontal and vertical scale bars represent 50 s and 2 kb, respectively.

We next tested the hypothesis that unlabeled UV-DDB may increase the rate of AAG dissociation from abasic (THF) sites using single molecule techniques. In these experiments AAG WT was labeled with 605 nm quantum dot (Qdot) using an antibody sandwich approach (32) in which a primary mouse anti-His antibody was bound to a His-tagged AAG WT protein, which was then conjugated to a goat anti-mouse secondary antibody-coated 605 nm Qdots (Figure 4A). Qdot-labeled AAG WT was observed using oblique angle TIRF microscopy and movies were obtained at 10 frames/s in the absence or presence of unlabeled UV-DDB on DNA tightropes containing one abasic (THF) site every 2 kb (Materials and Methods) for a total of 5 min, and the number of Qdot-labeled proteins dissociating during that time was noted. These movies are converted to kymographs showing position the *Y*-axis and time on the *X*-axis (Figure 4G and I). Time streaks showing the position on the DNA (*Y*-axis) over time (*X*-axis) are shown in the kymographs. Non-motile molecules show a straight line in the kymographs, whereas moving particles displace jagged traces (changes in the *Y*-position) over time. AAG WT displayed 3-fold increase in the frequency of dissociation (during the 5-min observation window) when the same amount of UV-DDB was added (Figure 4B, F–I, Supplementary Movies S1 and S2). Motile and persistent indicate that AAG is searching more efficiently in the presence of UV-DDB but has not dissociated in our time frame (5 min), whereas motile and dissociated suggest that AAG moving to search for damage and then dissociated to find another damage site with help of UV-DDB. Addition of equimolar UV-DDB to AAG WT decreases the half-life of AAG from 5300 to 644 s (Figure 4C). These data indicate that UV-DDB binding to abasic sites may help facilitate the dissociation of AAG WT bound to abasic sites. Addition of UV-DDB also induced AAG to undergo one-dimensional diffusion on DNA (Figure 4B and I), which can be observed in the kymographs as deviation from a straight line indicating motion.

Diffusion behavior of each motile AAG molecule was further analyzed by characterization of its anomalous diffusion exponent (α factor) and diffusion coefficient (D) in Figure 4D and E. The mean α factor of AAG only and AAG + 1× UV-DDB is 1.24 ± 0.28 and 1.11 ± 0.29, and its diffusion coefficient is (2.11 ± 0.80) × 10^−2^ μm^2^/s and (2.37 ± 0.85) × 10^−2^ μm^2^/s, respectively. These data suggest that AAG slides or hops along the DNA using random diffusion and are consistent with the two DNA-binding state modes of AAG reported previously (33). Compared to AAG alone, we found that the addition of 1× UV-DDB, while increasing the total motile number of AAG molecules, did not alter the relative rates of diffusion for motile AAG molecules (Supplementary Figure S3).

### UV-DDB can co-localize with AAG on DNA containing abasic sites

**Figure 5.**
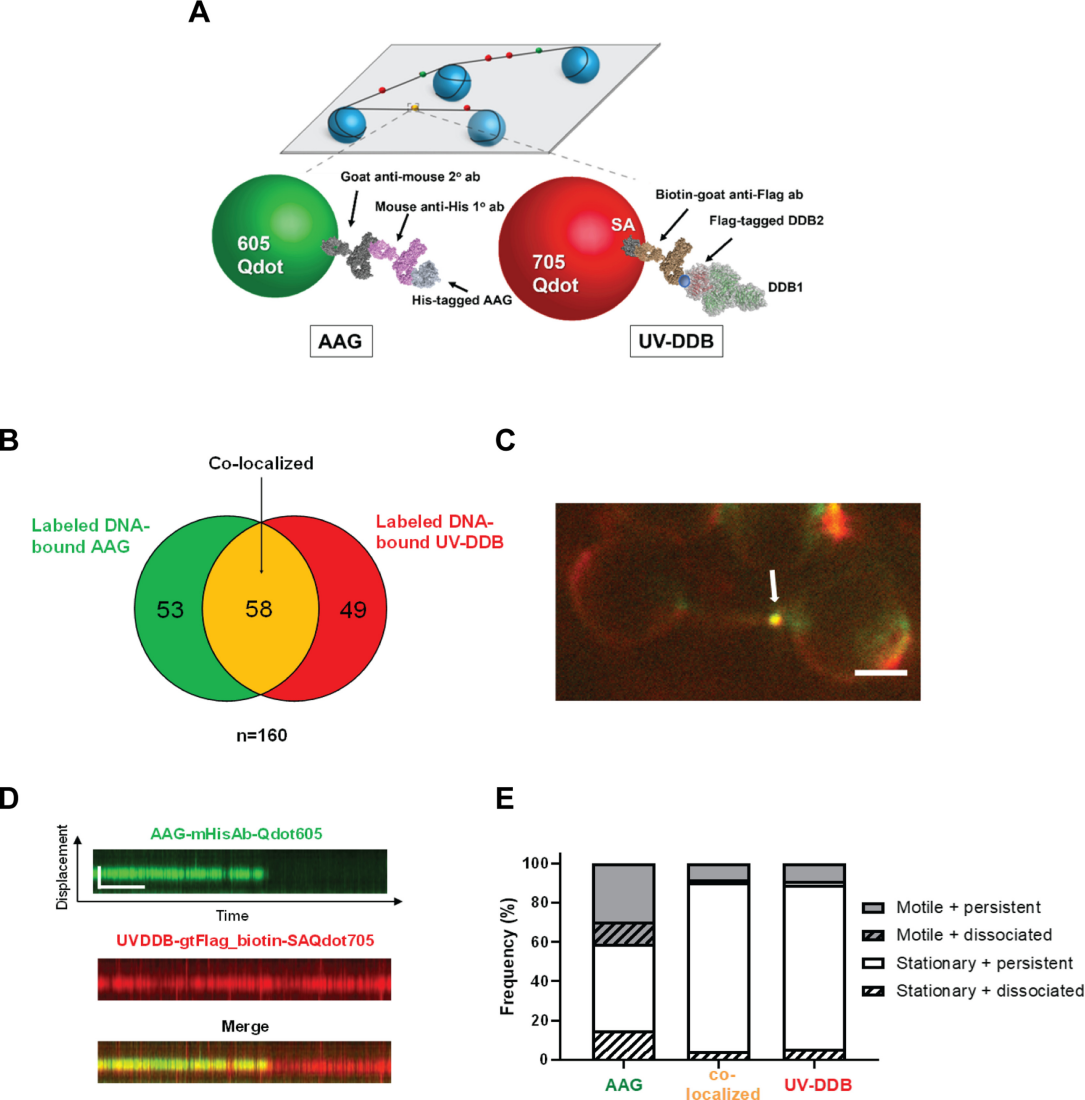
Single molecule co-localization of UV-DDB with AAG on abasic DNA tightropes. (**A**) Schematic of the DNA tightrope assay. Long DNA substrates with abasic sites every 2 kb were suspended between 5 μm poly-L-lysine coated silica beads. Anti-His primary antibody was used to link the His-tagged AAG to the 605Qdot. Biotin conjugated anti-Flag primary antibody was used to link Flag-tagged UV-DDB to streptavidin-coated 705Qdot. Uniquely labeled AAG and UV-DDB were observed interacting on abasic DNA tightropes in real time and their behavior and frequency of co-localization was recorded. Qdots are shown as a model and are not to scale. (**B**) Venn diagram showing number of proteins that co-localized (yellow) on abasic (THF) tightropes or were observed separately for 605Qdot-labeled AAG (green) with 705Qdot-labeled UV-DDB (red) in the dual-color assay. (**C**) Image of co-localized (yellow) Qdot-labeled AAG (green) and UV-DDB (red) on abasic (THF) tightrope suspended between beads. Scale bar represents 2.5μm. Arrow points to co-localized particle. (**D**) Kymograph of co-localized AAG and UV-DDB. Top, AAG (green); middle, UV-DDB (red); bottom, merged (yellow). Horizontal and vertical scale bars represent 50 s and 2 kb, respectively. (**E**) Stacked bar graph showing the fraction of motile (gray) versus stationary (white) and persistent (solid) versus dissociating (diagonal lines). Results obtained with individual and co-localized particles. (AAG: AAG behavior in the presence of UV-DDB; Co-localized: co-localized AAG-UV-DDB complex, UV-DDB: UV-DDB behavior in the presence of AAG). Data re-plotted as a sub-set of Figure 4B.

Since both AAG and UV-DDB are capable of binding to abasic sites individually or together, we sought to further investigate their potential interactions on the DNA containing abasic sites. To do this, we used an orthogonal labeling strategy for direct dual-color fluorescence imaging of His-tagged AAG, labeled with 605 nm Qdots and Flag-tagged UV-DDB, labeled with a biotinylated goat anti-Flag primary antibody and streptavidin-coated 705 nm Qdots, as described (Figure 5A). Control experiments indicated that there is no exchange of Qdots between AAG and UV-DDB.

Qdot-labeled AAG was first injected into a flow cell containing DNA tightropes with one abasic site (THF) every 2 kb, and then Qdot labeled UV-DDB was injected. After injection, all flow was stopped. Despite the transient nature of AAG and UV-DDB binding to damaged DNA, we found that colocalization of AAG and UV-DDB accounted for 36.3% of all particles (Figure 5B). The kymographs of merged channels (Figure 5D) showed colocalization of green and red signals, indicating specific binding of AAG with UV-DDB. Additional kymographs are shown in Supplementary Figure S4A–H and Supplementary Movie S3. Some of these colocalized molecules (Supplementary Figure S4A, D, E and H) were found to diffuse on the DNA together, suggesting direct interactions on DNA. Interestingly at colocalized molecules, we observed a significant decrease of the yellow colocalized signal over time due to the disappearance of the green AAG signal indicating that UV-DDB helps to dissociate AAG from abasic sites (Figure 5C and D), Also note that UV-DDB helps to induce mobility and increase dissociation of AAG (Figure 5E). Taken together, these data suggest that UV-DDB can associate and migrate together with AAG on DNA and stimulate AAG turnover.

### Following the kinetics of AAG interaction on hypoxanthine moieties

**Figure 6.**
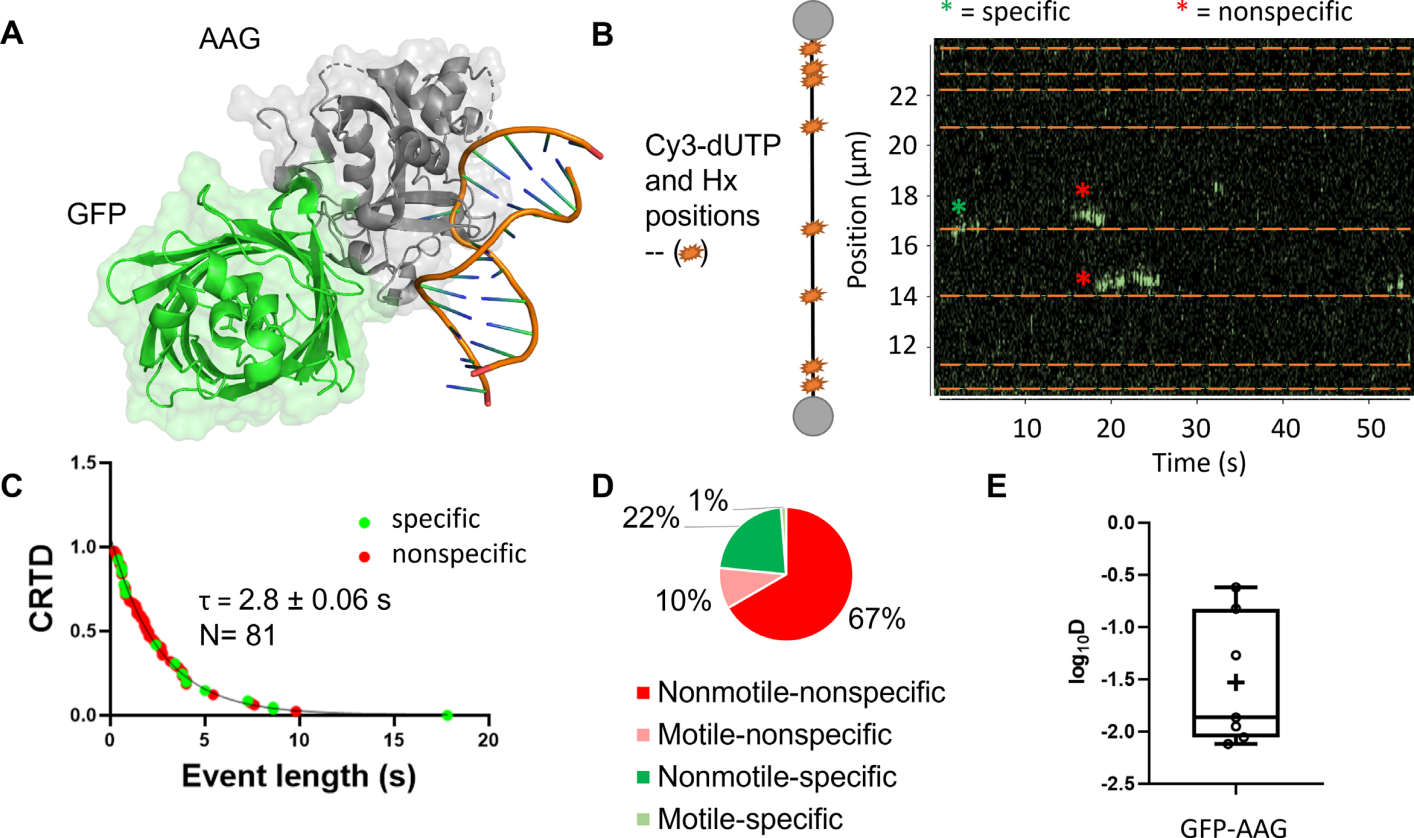
SMADNE characterization of GFP-AAG with hypoxanthine modified DNA. (**A**) The structure of alkyladenine glycosylase (AAG) with an N-terminal turbo GFP tag. Structures taken from PDB codes: 1F4R and 4KW4. (**B**) Nick translation allows for the simultaneous incorporation of Cy3-dUTP and dITP (inosine triphosphate, the nucleotide form of Hx). Cy3 incorporation positions were determined via fluorescence and are shown as orange stars and dotted lines. In the example kymograph, GFP-AAG binding events are shown in green, with off-target events marked with a red asterisk and on-target events with a green asterisk. (**C**) The cumulative residence time distribution of all GFP-AAG events, fitting to a single-exponential with a lifetime of 2.8 ± 0.06 s. (**D**) The distribution of events that occurred on the Cy3 labeled sites (interact) as well as which were motile and nonmotile. (**E**) A plot of the diffusivity for motile GFP-AAG events. The mean diffusivity for these events was 6.9 ± 3.2 × 10^−2^ μm^2^/s.

We sought to extend our studies of damage detection by AAG on abasic sites to substrates with hypoxanthine substrates. Tightrope imaging of Qdot-labeled proteins does not easily allow the analysis of transient (seconds) protein interactions with DNA, nor allowed the positions of the abasic sites to be precisely known, and we thus combined two novel approaches to follow AAG interacting with hypoxanthine moieties in lambda DNA. First, to create hypoxanthine sites within lambda DNA, dITP was incorporated at 10 nick sites created by the nickase Nt.BspQI via nick translation with Pol I. Cy3-labeled dUTP was also incorporated at the same time to provide fluorescent fiducial markers for the positions of hypoxanthine moieties. Second, we used a newly developed approach, single-molecule analysis of DNA-binding proteins from nuclear extracts (SMADNE) from cells transfected with a plasmid expressing GFP-tagged AAG (Figure 6A) (28). These nuclear extracts were prepared and diluted by a factor of 3:10 and flowed into a microfluidic chamber of a LUMICKS C-trap. In this approach two 4-micron beads coated with streptavidin are first captured in two laser traps, biotinylated lambda DNA containing hypoxanthine is flowed into the chamber, and a single DNA molecule is tethered between the beads. After washing the tethered DNA in chamber 3, nuclear extract containing GFP-AAG at an average concentration of 0.3 nM was flowed into channel 4, the flow was stopped, the DNA substrate was moved into the nuclear extract, and binding events along the DNA were recorded (Figure 6B). The fluorescent fiducial marker and hypoxanthine positions were measured by briefly toggling a 562 nm laser on and off, and events with GFP-AAG were collected by exciting with a 488 nm laser. Cumulative residence time distribution analysis of all events observed revealed a binding lifetime with GFP-AAG to be 2.8 ± 0.06 s (Figure 6C). Of these events, a majority of them were brief sampling events that occurred on sites without the DNA damage (77%) but 23% of events did colocalize with the damage sites (Figure 6D). About 11% of events were motile, a similar fraction as was observed by AAG on abasic-site containing DNA, and of those motile events, the average linear diffusivity was (6.9 ± 3.2) × 10^−2^ μm^2^/s (Figure 6E). This faster diffusivity is due to the presence of the smaller tag compared to the previous tightrope data using Qdot labeling.

### UV-DDB and AAG co-localize at MMS-induced damage in cells

**Figure 7.**
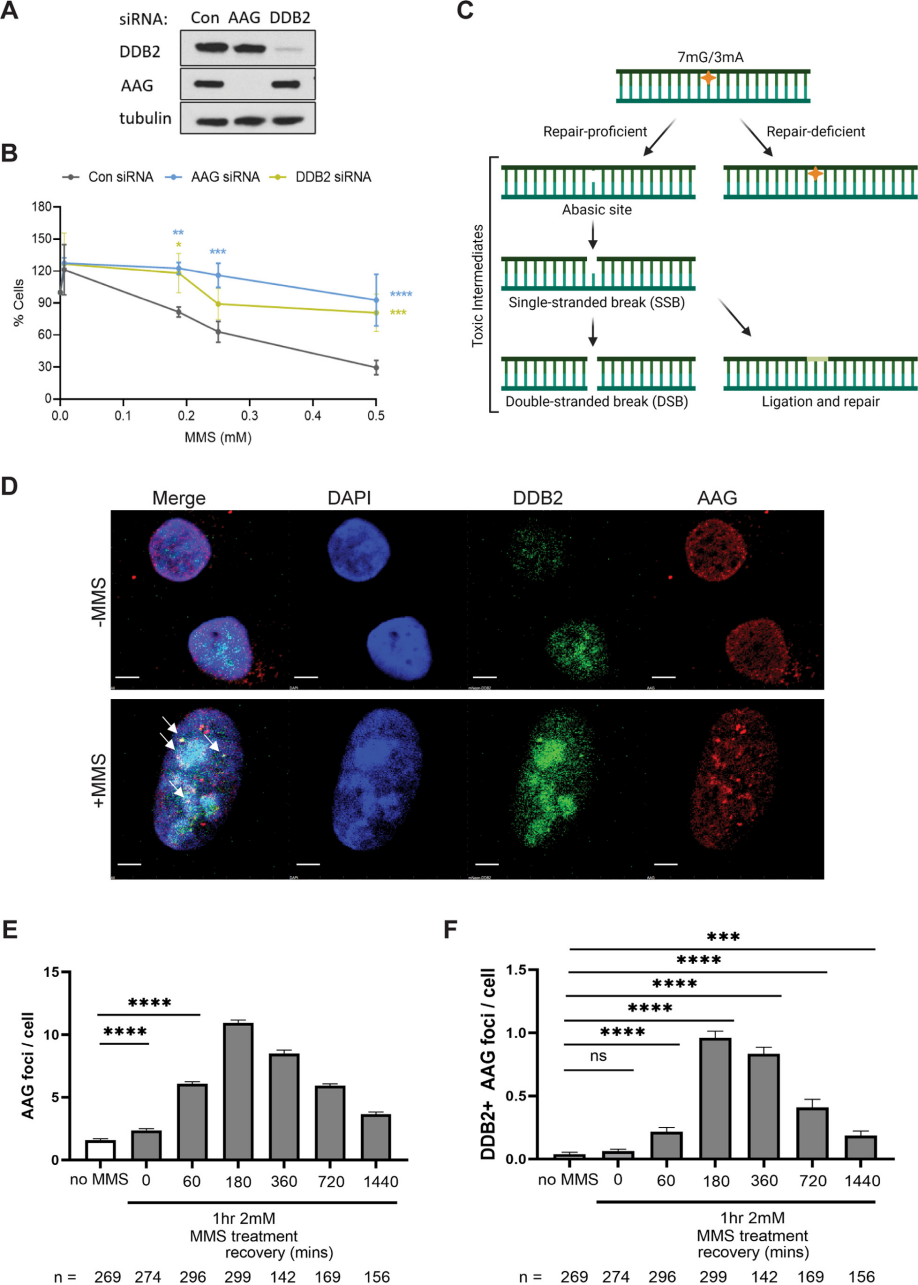
DDB2 or AAG KD cells are more resistant to MMS damage, and UV-DDB and AAG co-localize at MMS-induced damage in cells**. (A**) Western blot showing AAG and DDB2 protein levels, 48 hours post transfection with respective siRNAs. (**B**) Cells were treated with indicated concentrations of MMS and allowed to recover for 8 days. Colonies were stained with crystal violet and counted. Data show one representative experiment (performed in triplicate), mean ± SD. Two-way ANOVA was performed for statistical analysis: *P < 0.05, **P < 0.01, ***P < 0.001, ****P < 0.0001. (**C**) Schematic explaining the toxic intermediates formed during repair of MMS-induced DNA damage. (**D**) U2OS cells stably expressing mNeonGreen-DDB2 were treated with 2 mM MMS for 1 h. After MMS treatment, cells were washed and recovered in fresh medium for 0 to 24 h. Top panel shows that, without MMS treatment, AAG and DDB2 signal are evenly distributed in the nuclei. Scale bar = 5 microns. Bottom panel shows that, 3-h post MMS treatment, AAG foci were visualized, and some AAG foci were colocalized with DDB2 foci (pointed by yellow arrows). Scale bar = 1 micron. (**E**) Quantification of AAG foci in (D). (**F**) Quantification of DDB2 colocalized AAG foci in (D). ‘*n*’ shows the number of cells in each condition. Statistics was calculated with Prism 9.1.1 based on three experiments. Error bars show mean ± SEM. (ns: *P* > 0.05, *: *P* < 0.05, **: *P* < 0.01, ***: *P* < 0.001, ****: *P* < 0.0001).

Previous studies of MMS sensitivity in mammalian cells have shown a complex phenotype in which BER intermediates rather than the alkylated bases are more toxic to cells (34). U2OS cells depleted for either AAG or DDB2 by siRNA transfection were treated with MMS for an hour (0–0.5 mM) and surviving colonies were counted 8 days later (Figure 7A and B). Knockdown of either AAG or DDB2 caused an increased resistance to MMS treatment, suggesting that processing of MMS damage by these two proteins is toxic to the cells. This is consistent with previous reports suggesting that toxic intermediates formed during BER could be more lethal than the initial base damage (Figure 7C) (35,36). It has been previously suggested that BER proteins form discrete foci after MMS treatment (37). We therefore wanted to next determine if DDB2 formed discrete foci after MMS damage. In initial experiments, U2OS cells were transfected with DDB2-mCherry, and treated with MMS for 30 min at 0.5 or 1 mM MMS. We observed a dose-dependent increase in DDB2-mCherry foci, as well as γ-H2AX foci; however, no co-localization was observed (Supplementary Figure S5). These data suggest that DDB2 localizes to the base damage and not the double-strand breaks (DSBs) formed by MMS. We then used U2OS cells stably expressing mNeonGreen-DDB2 at about 3-fold higher expression than the endogenous DDB2 (Supplementary Figure S6), which were treated with 2 mM MMS for 1 h and the cells were then collected at indicated timepoints post treatment. Antibodies to endogenously expressed AAG showed discrete foci peaking at 3 h. Using confocal microscopy and direct mNEONGreen imaging, we observed significant DDB2 colocalization with AAG foci after 3 h following MMS treatment (Figure 7 D–F and Supplementary Figures S6 and S7, Supplementary movies S4 and S5). Together these results suggest that UV-DDB participates with AAG in the processing of alkylation damage resulting from MMS.

## DISCUSSION

We have previously shown that UV-DDB helps facilitate the processing of 8-oxoG by OGG1 and MUTYH (17,18). We also showed that UV-DDB stimulates APE1 and gap-filling by DNA polymerase β (17). In this present study we assessed whether UV-DDB participates with AAG in the removal of ϵA or Hx moieties. UV-DDB was found to recognize ϵA:T and Hx:T pairs with affinities similar to a duplex DNA containing a TT CPD, and was able to stimulate AAG activity 4- to 5-fold on DNA duplexes containing these lesions. Single molecule analysis and native PAGE showed that UV-DDB can co-localize and displace AAG from abasic sites. A new single molecule approach using fluorescently tagged AAG from nuclear extracts was able to show specific binding to Hx moieties embedded in lambda DNA. Cell experiments showed that loss of either DDB2 or AAG caused increased resistance to MMS damage. Finally, we observed colocalization of AAG and DDB2 following MMS treatment. Together these results combined with our previous studies indicate that UV-DDB recognizes a wide range of lesions stimulating BER (17,18,31).

**Figure 8.**
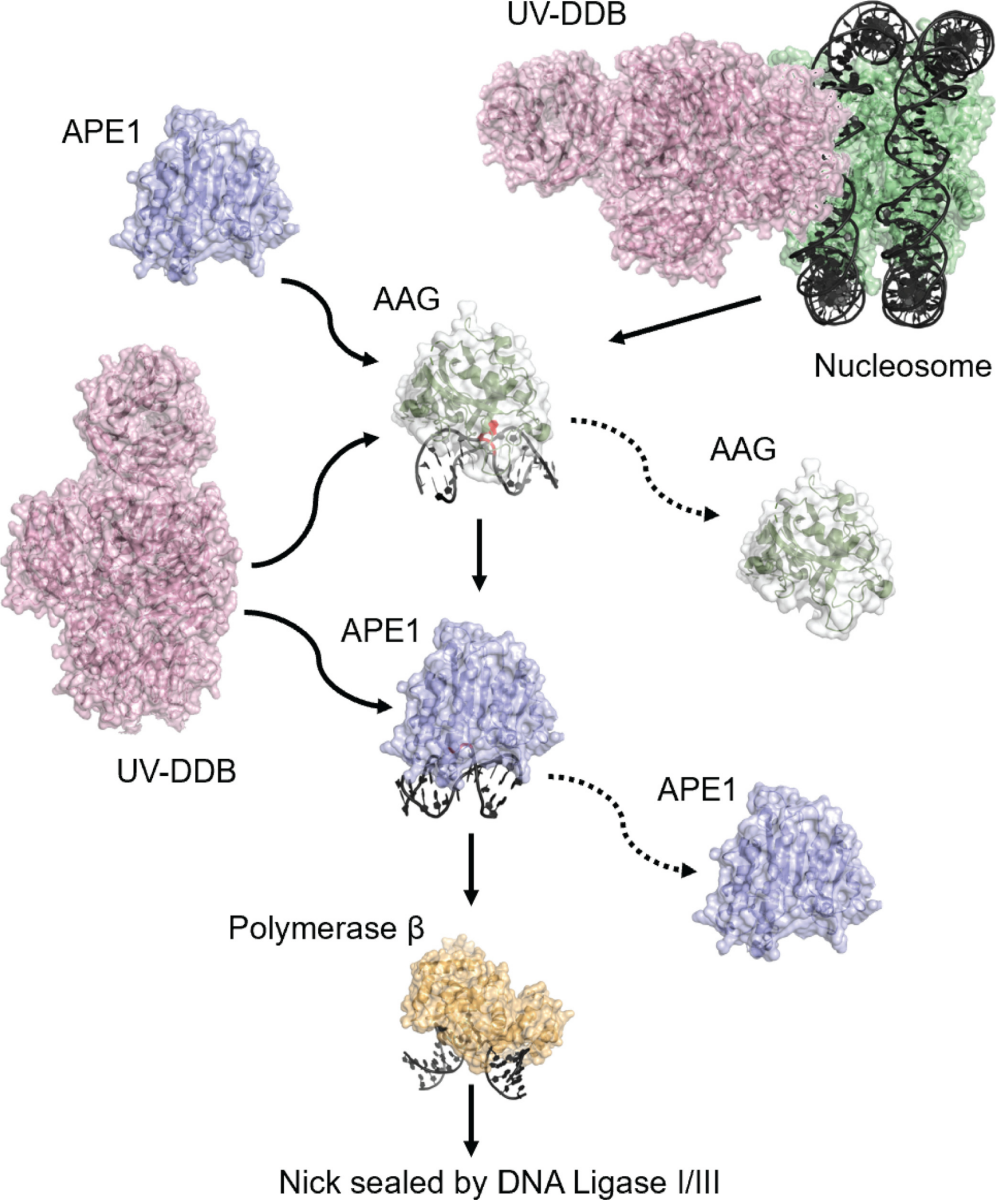
Schematic representation of the proposed BER pathway including UV-DDB. Proposed working model. Schematic representation of the proposed BER pathway including UV-DDB is illustrated. UV-DDB appears to be rapidly recruited to damaged sites in chromatin and help facilitate processing by AAG. Biochemical and single molecule data suggest that UV-DDB transiently associates with AAG at abasic sites to increase their turnover and stimulated BER (see text for description).

Figure 8 provides a working model that is consistent with the known literature and the results presented in this study. DNA in mammalian cells in organized into chromatin with the simplest repeat is the nucleosome core particle (NCP) consisting of 147 bp of DNA wrapped around an octameric complex consisting of two molecules each of H2A, H2B, H3 and H4. Several groups have shown that glycosylases have difficulty in processing base damage contained within nucleosome core particles (38–46). For example, work by Sarah Delaney *et al.* showed AAG has a lower ability to excise ϵA lesions within 601 positioned nucleosomes as compared to DNA duplexes, and certain sites that are poorly solvent accessible are poor substrates (47,48). These results are consistent with work by Wyrick *et al.* who found that global repair of MMS damage from the budding yeast was more rapid in nucleosome free regions as compared to nucleosome containing sites of the genome (49,50). Thoma *et al.* used cryo-EM to study UV-DDB’s interaction with a nucleosome containing defined damage site at various positions within the nucleosome. UV-DDB was shown to shift the DNA register, altering the nucleosome architecture by three base pairs to allow access to occluded sites (19). Furthermore, we have shown that DDB2 is necessary and sufficient to decompact chromatin around sites of 8-oxoG (51). Thus, our new data and these data with 8-oxoG suggest that UV-DDB might be the first responder to alkylation damage and deamination products of A and help displace nucleosomes from DNA containing alkylation damage.

While AAG shows less affinity for abasic sites than other glycosylases, we believe that the low rate of turnover of AAG we and others have observed is attributable to its ability to bind to abasic sites with equal affinity as ϵA or Hx (15,16). Our single molecule tightrope analysis using Qdot labeled AAG binding to THF containing DNA suggested long lived complexes which can be readily displaced by UV-DDB (Figures 4 and 5). Since these tightrope experiments were designed to study product release by AAG, we developed an innovative and robust single-molecule characterization for AAG searching for hypoxanthine lesions in the context of nuclear extracts, an environment that more closely mimics the conditions of the nucleus than that of purified proteins. We found that AAG remains stationary at sites of Hx incorporation, but has increased linear diffusion while binding non-specifically to DNA. While the diffusivity of events seemed relatively consistent between the approaches, the lifetime with the SMADNE approach was much reduced. This may be due to non-specific binding to DNA by AAG, which samples DNA briefly could also be detected on this new C-trap platform and were not readily observable with the tightrope assay which detects longer lived events. This shorter lifetime could also be due to other proteins in the nuclear extract such as UV-DDB or APE1 assisting with the dissociation of AAG. Finally, since Qdots do not photobleach thus providing access to longer dwell times.

Our ability to show complex formation between AAG and UV-DDB both biochemically (Figure 3) and at the single molecule level on THF containing DNA in which about 37% of the AAG and UV-DDB molecules that were observed formed transient complexes suggests that both proteins might be able to interact during processing of alkylated bases or deamination products. Direct interactions for these two proteins could not be detected off the DNA and alphafold multimer (52) did not predict an interaction. AAG’s mobility on DNA increases in the presence of UV-DDB and that this type of motion was predicted to occur by Samson and colleagues (33). Thus, AAG may slide along the major groove using Tyr162 as a wedge residue to interrogate the bases for damage, has been shown by Wallace and colleagues for wedge residues in the *Escherichia coli* glycosylases Fpg (F111), Nei (F111) and Nth (L81) (27,53).

Our cellular experiments showing discrete foci of AAG and DDB2, with some co-localization, peaking at 3 h after MMS treatment further supports the concept that AAG and UV-DDB cooperate in processing of alkylated bases (Figure 7). Foci formation of other BER proteins, including APE1 (54), XRCC1 (37) and PCNA (55) have been observed previously after MMS damage, and has also been reported after hydrogen peroxide damage in mammalian cells (56).

We found that cells lacking DDB2 were more resistant to killing by MMS than their WT counterparts. It is interesting to note that mice either completely deficient or haploid for *Ddb2* expression, suffer premature death due to high numbers of spontaneous tumors (57,58). Also, it was shown in an azoxymethane/dextran sulfate-induced mouse model of GI cancer thatmice deficient in DDB2 developed a greater number and larger tumors than their WT counterparts (59). These observations when combined with the findings presented in this present study, suggest that UV-DDB and especially DDB2 might play a wider role in damage recognition and processing of endogenous DNA damage.

## DATA AVAILABILITY

All raw data is available and can be released upon request from the corresponding author, vanhoutenb@upmc.edu.

## Supplementary Material

gkac1145_Supplemental_Files

## Notes

Present address: Zhou Zhong, LUMICKS, Waltham, MA, USA.
