# Supplementary material for: Cooperative interaction between AAG and UV-DDB in the removal of modified bases
Source: Nucleic Acids Res. 2022 Dec 13;50(22):12856–71. doi: 10.1093/nar/gkac1145 (PMC9825174; doi:10.1093/nar/gkac1145)
Supplement: gkac1145_Supplemental_Files [file gkac1145_supplemental_files.zip › gkac1145_Supplementary figures_Edited_12_09_22.pdf]

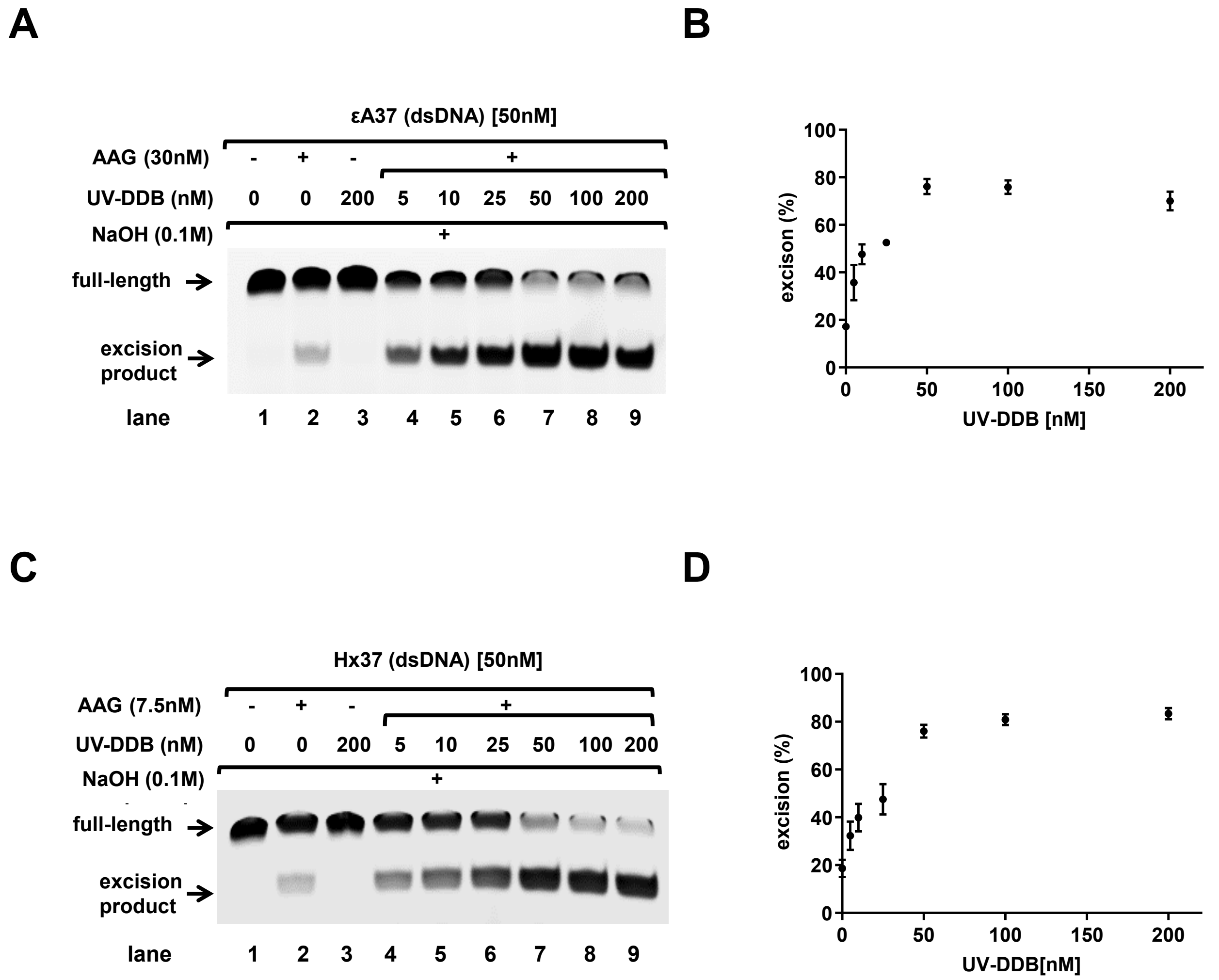

Figure S1. AAG activities in the absence or presence of UV-DDB. Related to Figure 2.

(A) Effect of UV-DDB concentration on stimulation of AAG excision. εdA37 dsDNA was incubated with AAG and/or increasing amounts of UV-DDB at 37°C for 2hrs and separated by denaturing polyacrylamide electrophoresis. **(B)** Quantification of (A). Percent of total DNA that was excised by AAG plotted as a function of UV-DDB concentration. Data shown as the mean of three experiments ± SD. **(C)** Effect of UV-DDB concentration on stimulation of AAG excision. Hx37 dsDNA was incubated with AAG and/or increasing amounts of UV-DDB at 37°C for 2hrs and separated by denaturing polyacrylamide electrophoresis. **(D)** Quantification of (C). Percent of total DNA that was excised by AAG plotted as a function of UV-DDB concentration. Data shown as the mean of three experiments ± SD.

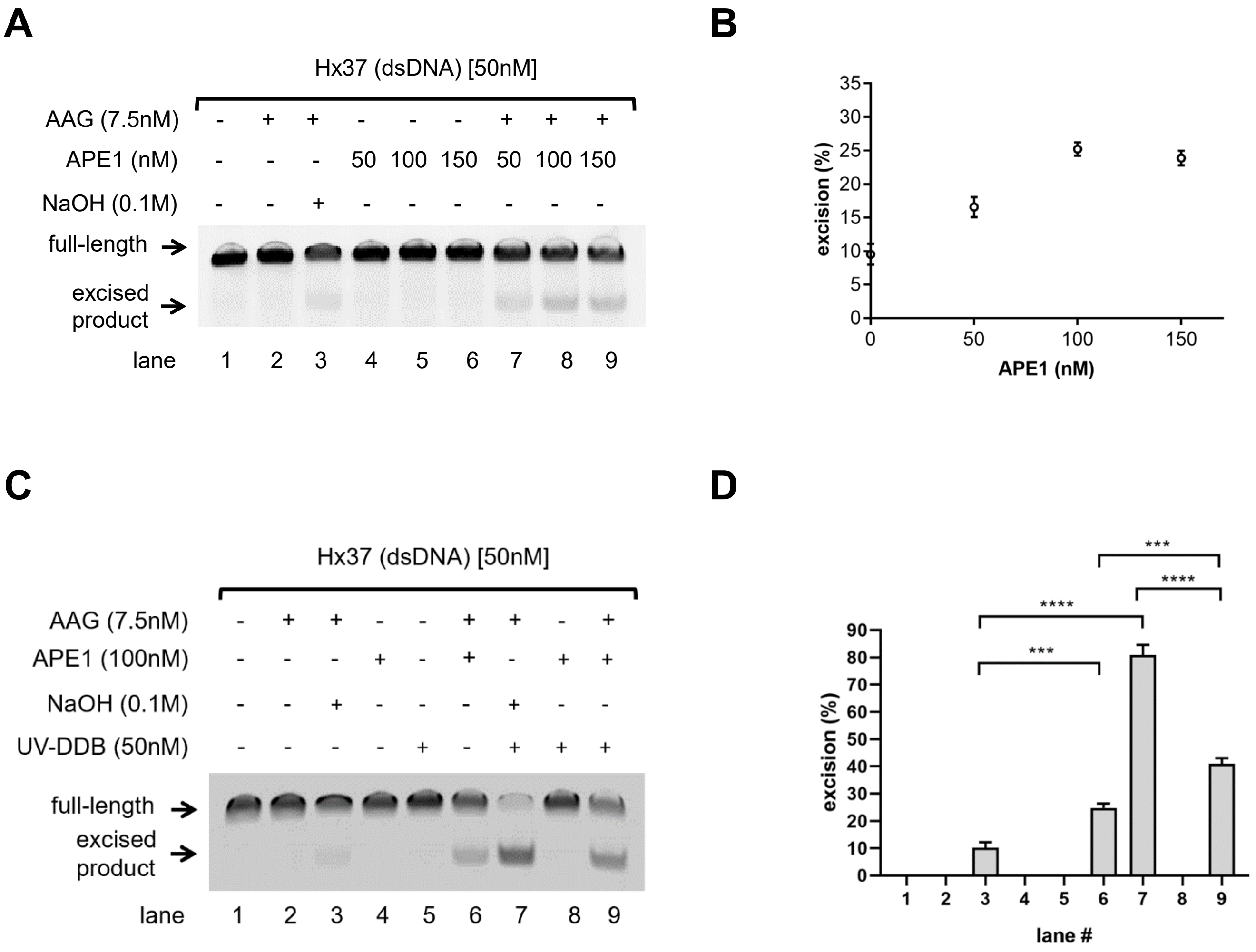

Figure S2. Effect of APE1 and UV-DDB on stimulation of AAG, Related to Figure 2.

(A) Effect of APE1 concentration on stimulation of AAG excision. Hx37 was incubated with AAG and/or increasing amount of APE1 for 1hr at 37°C. The reaction was immediately stopped by adding 2X loading dye followed by heating 95°C for 5mins then quickly chilling on ice for 5mins. **(B)** Quantification of (A). Percent of total DNA that was excised by AAG plotted as a function of APE1 concentration. Data shown as the mean of three experiments ± SD. **(C)** Effect of APE1 or UV-DDB on stimulation on AAG excision. Hx37 was incubated with AAG only, AAG + APE1, AAG +UV-DDB or AAG + APE1 + UV-DDB for 1.5hr at 37°C and separated by denaturing polyacrylamide electrophoresis. **(D)** Quantification of (C). Percent of total DNA that was excised by AAG plotted as lane number. Data shown as the mean of three experiments ± SD. (*** P<0.001, **** P<0.0001).

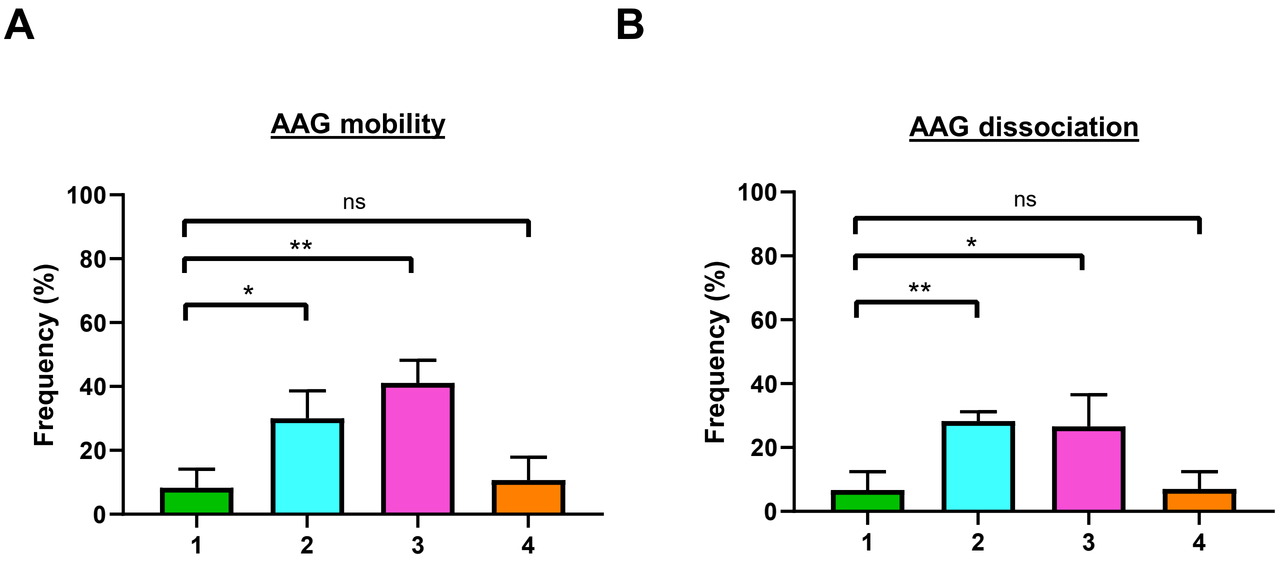

1. AAG-mHis-605Qdot
2. AAG-mHis-605Qdot
   in the presence of 1x unlabeled UV-DDB
3. AAG-mHis-605Qdot
   in the presence of UV-DDB-gFlagb-705SAQdot (not co-localized)
4. AAG-mHis-605Qdot
   in the presence of UV-DDB-gFlagb-705SAQdot (co-localized)

Figure S3. Single molecule analysis reveals UV-DDB stimulates AAG by facilitated mobility and dissociation. Related to Figures 4 & 5.

(A) Mobility of 605Qdot-labeled AAG on DNA tightropes containing abasic sites (THF) in the absence (1) or presence (2,3 & 4) of UV-DDB. Bar graph data shown as means ± SD with three (1,2) or four (3,4) independent experiments. (* $p<0.1$, ** $p<0.01$, by two-tailed Student's t test). **(B)** Dissociation of 605Qdot-labeled AAG on DNA tightropes containing abasic sites (THF) in the absence (1) or presence (2,3 & 4) of UV-DDB. Bar graph data shown as mean ±SD with three (1,2) or four (3,4) independent experiments. (* $p<0.1$, ** $p<0.01$, by two-tailed Student's t test)

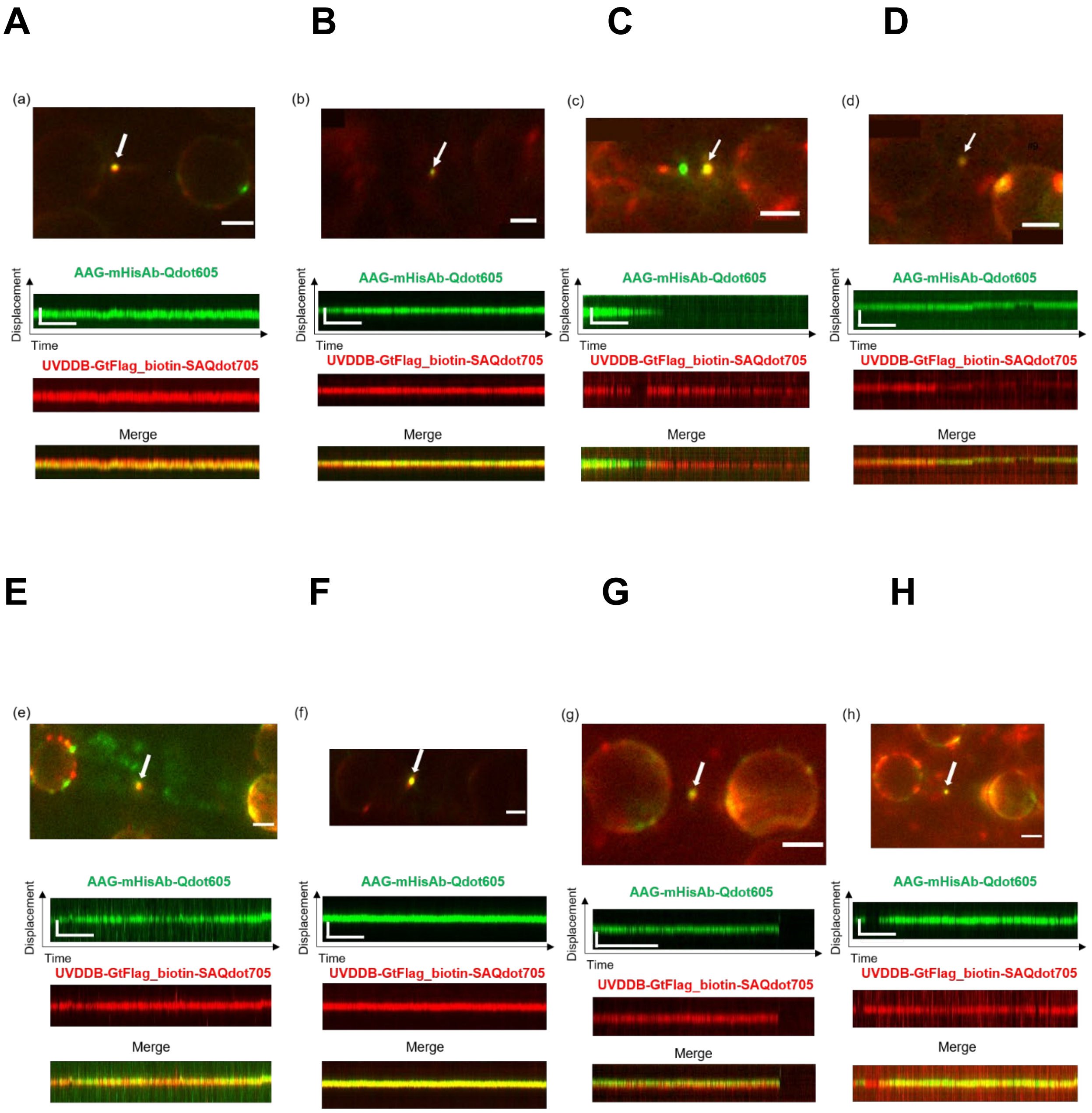

Figure S4. DNA tightrope assay showing co-localization of UV-DDB and AAG, Related to Figure 5.

(A-H), Additional still frames and corresponding kymographs of co-localized AAG and UV-DDB (AAG: green, UV-DDB: red, and merge: yellow). Top, scale bar represents 2.5 μm; arrows point to co-localized particles. Bottom, horizontal and vertical scale bars represent 50 s and 2 kb, respectively.

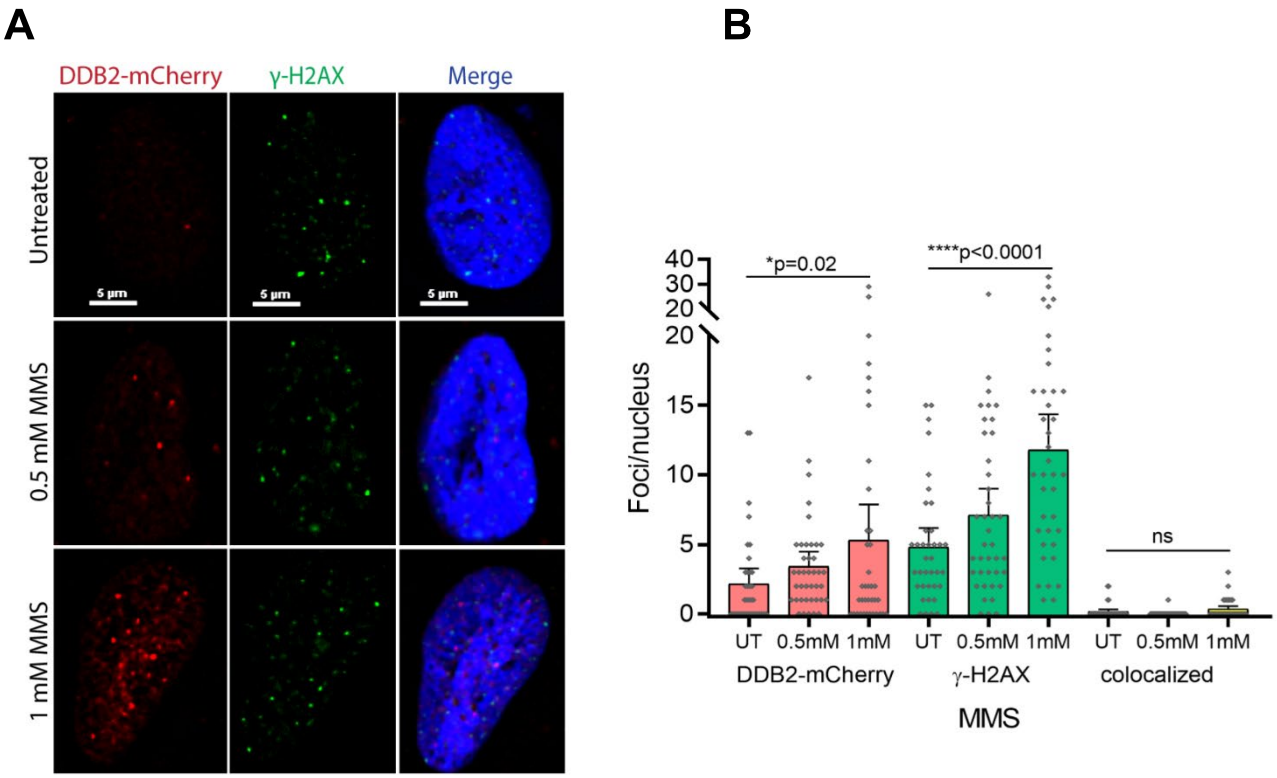

Figure S5. UV-DDB and AAG co-localize at MMS-induced damage in cells. Related to Figure 7. (A) U2OS cells were transiently transfected with DDB2-mCherry. 48 hours post transfection, cells were treated with MMS for 30 minutes before staining for mCherry and γ-H2AX. **(B)** Quantification of foci formed per nucleus. 40 cells were scored for each condition. Data represents mean ± SD. One-way ANOVA was performed for statistical analysis: ns: not significant, *p<0.05, ****p<0.0001.

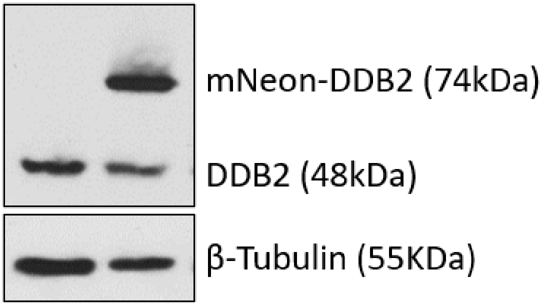

Figure S6. Western blot of DDB2-mNeonGreen. Related to Figure 7. Cells stably expressing mNeonGreen-DDB2 were lysed and run on an SDS-PAGE to determine protein levels. There was an ~3-fold overexpression of mNeonGreen- DDB2 compared to endogenous DDB2.

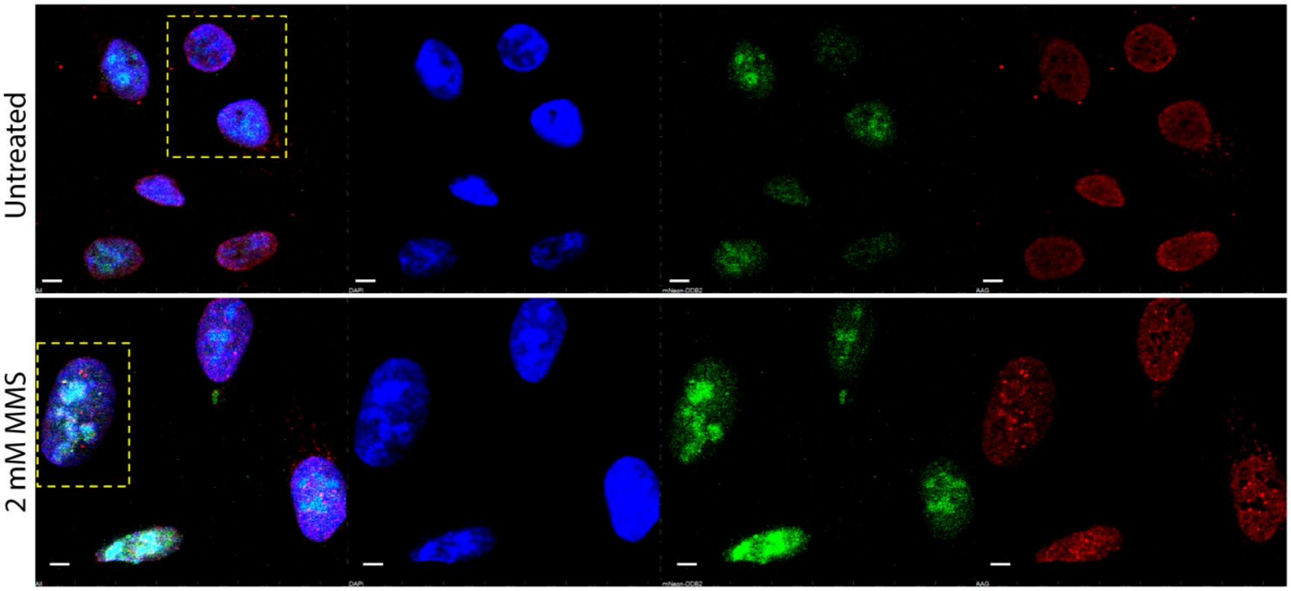

Figure S7. Immunofluorescence field used in Figure 7. U2OS cells stably expressing mNeonGreen-DDB2 were treated with 2mM MMS for 1 hour. Representative images of cells, 3 hours post treatment, is shown. Yellow boxes indicate the cells used in Figure 7.

Descriptions of Videos

Video 1: 605 nm-Qdot labelled-AAG a DNA tightrope containing abasic sites in the presence of unlabeled UV-DDB corresponding to **Figure 4F**. Data were collected at 11.38fps and are played back at 30fps.

Video 2: 605 nm-Qdot labelled-AAG induced motion and dissociation from a DNA tightrope containing abasic sites induced by unlabeled UV-DDB corresponding to **Figure 4H**. Data were collected at 11.38fps and are played back at 30fps.

Video 3: Transient **c**o-localization and displacement of 605 nm-Qdot labelled-AAG with 705 nm Qdot labelled-UV-DDB. Molecule complex on left goes from yellow to red showing displacement of AAG. **Figure 5D**. Data were collected at 1.74fps and are played back at 12fps.

Video 4: Confocal Z stack of mNEON-DDB2 and AAG in untreated cells, image is shown in **Figure 7 and S7**.

Video 5: Confocal Z stack of mNEON-DDB2 and AAG images after a 3 hour period recover from MMS treated (2 mM, 1hr) cells, image is shown in **Figure 7 and S7**.
